# Objects of consciousness

**DOI:** 10.3389/fpsyg.2014.00577

**Published:** 2014-06-17

**Authors:** Donald D. Hoffman, Chetan Prakash

**Affiliations:** ^1^Department of Cognitive Sciences, University of CaliforniaIrvine, CA, USA; ^2^Department of Mathematics, California State UniversitySan Bernardino, CA, USA

**Keywords:** consciousness, quantum theory, Markov chains, combination problem, geometric algebra

## Abstract

Current models of visual perception typically assume that human vision estimates true properties of physical objects, properties that exist even if unperceived. However, recent studies of perceptual evolution, using evolutionary games and genetic algorithms, reveal that natural selection often drives true perceptions to extinction when they compete with perceptions tuned to fitness rather than truth: Perception guides adaptive behavior; it does not estimate a preexisting physical truth. Moreover, shifting from evolutionary biology to quantum physics, there is reason to disbelieve in preexisting physical truths: Certain interpretations of quantum theory deny that dynamical properties of physical objects have definite values when unobserved. In some of these interpretations the observer is fundamental, and wave functions are compendia of subjective probabilities, not preexisting elements of physical reality. These two considerations, from evolutionary biology and quantum physics, suggest that current models of object perception require fundamental reformulation. Here we begin such a reformulation, starting with a formal model of consciousness that we call a “conscious agent.” We develop the dynamics of interacting conscious agents, and study how the perception of objects and space-time can emerge from such dynamics. We show that one particular object, the quantum free particle, has a wave function that is identical in form to the harmonic functions that characterize the asymptotic dynamics of conscious agents; particles are vibrations not of strings but of interacting conscious agents. This allows us to reinterpret physical properties such as position, momentum, and energy as properties of interacting conscious agents, rather than as preexisting physical truths. We sketch how this approach might extend to the perception of relativistic quantum objects, and to classical objects of macroscopic scale.

## Introduction

The human mind is predisposed to believe that physical objects, when unperceived, still exist with definite shapes and locations in space. The psychologist Piaget proposed that children start to develop this belief in “object permanence” around 9 months of age, and have it firmly entrenched just 9 months later (Piaget, 1954). Further studies suggest that object permanence starts as early as 3 months of age (Bower, 1974; Baillargeon and DeVos, 1991).

Belief in object permanence remains firmly entrenched into adulthood, even in the brightest of minds. Abraham Pais said of Einstein, “We often discussed his notions on objective reality. I recall that on one walk Einstein suddenly stopped, turned to me and asked whether I really believed that the moon exists only when I look at it” (Pais, 1979). Einstein was troubled by interpretations of quantum theory that entail that the moon does not exist when unperceived.

Belief in object permanence underlies physicalist theories of the mind-body problem. When Gerald Edelman claimed, for instance, that “There is now a vast amount of empirical evidence to support the idea that consciousness emerges from the organization and operation of the brain” he assumed that the brain exists when unperceived (Edelman, 2004). When Francis Crick asserted the “astonishing hypothesis” that “You're nothing but a pack of neurons” he assumed that neurons exist when unperceived (Crick, 1994).

Object permanence underlies the standard account of evolution by natural selection. As James memorably put it, “The point which as evolutionists we are bound to hold fast to is that all the new forms of being that make their appearance are really nothing more than results of the redistribution of the original and unchanging materials. The self-same atoms which, chaotically dispersed, made the nebula, now, jammed and temporarily caught in peculiar positions, form our brains” (James, 1890). Evolutionary theory, in the standard account, assumes that atoms, and the replicating molecules that they form, exist when unperceived.

Object permanence underlies computational models of the visual perception of objects. David Marr, for instance, claimed “We … very definitely do compute explicit properties of the real visible surfaces out there, and one interesting aspect of the evolution of visual systems is the gradual movement toward the difficult task of representing progressively more objective aspects of the visual world” (Marr, 1982). For Marr, objects and their surfaces exist when unperceived, and human vision has evolved to describe their objective properties.

Bayesian theories of vision assume object permanence. They model object perception as a process of statistical estimation of object properties, such as surface shape and reflectance, that exist when unperceived. As Alan Yuille and Heinrich Bülthoff put it, “We define vision as perceptual inference, the estimation of scene properties from an image or sequence of images … ” (Yuille and Bülthoff, 1996).

There is a long and interesting history of debate about which properties of objects exist when unperceived. Shape, size, and position usually make the list. Others, such as taste and color, often do not. Democritus, a contemporary of Socrates, famously claimed, “by convention sweet and by convention bitter, by convention hot, by convention cold, by convention color; but in reality atoms and void” (Taylor, 1999).

Locke proposed that “primary qualities” of objects, such as “bulk, figure, or motion” exist when unperceived, but that “secondary properties” of objects, such as “colors and smells” do not. He then claimed that “… the ideas of primary qualities of bodies are resemblances of them, and their patterns do really exist in the bodies themselves, but the ideas produced in us by these secondary qualities have no resemblance of them at all” (Locke, 1690).

Philosophical and scientific debate continues to this day on whether properties such as color exist when unperceived (Byrne and Hilbert, 2003; Hoffman, 2006). But object permanence, certainly regarding shape and position, is so deeply assumed by the scientific literature in the fields of psychophysics and computational perception that it is rarely discussed.

It is also assumed in the scientific study of consciousness and the mind-body problem. Here the widely acknowledged failure to create a plausible theory forces reflection on basic assumptions, including object permanence. But few researchers in fact give it up. To the contrary, the accepted view is that aspects of neural dynamics—from quantum-gravity induced collapses of wavefunctions at microtubules (Hameroff, 1998) to informational properties of re-entrant thalamo-cortical loops (Tononi, 2004)—cause, or give rise to, or are identical to, consciousness. As Colin McGinn puts it, “we know that brains are the *de facto* causal basis of consciousness, but we have, it seems, no understanding whatever of how this can be so” (McGinn, 1989).

## Evolution and perception

The human mind is predisposed from early childhood to assume object permanence, to assume that objects have shapes and positions in space even when the objects and space are unperceived. It is reasonable to ask whether this assumption is a genuine insight into the nature of objective reality, or simply a habit that is perhaps useful but not necessarily insightful.

We can look to evolution for an answer. If we assume that our perceptual and cognitive capacities have been shaped, at least in part, by natural selection, then we can use formal models of evolution, such as evolutionary game theory (Lieberman et al., 2005; Nowak, 2006) and genetic algorithms (Mitchell, 1998), to explore if, and under what circumstances, natural selection favors perceptual representations that are genuine insights into the true nature of the objective world.

Evaluating object permanence on evolutionary grounds might seem quixotic, or at least unfair, given that we just noted that evolutionary theory, as it's standardly described, assumes object permanence (e.g., of DNA and the physical bodies of organisms). How then could one possibly use evolutionary theory to test what it assumes to be true?

However, Richard Dawkins and others have observed that the core of evolution by natural selection is an abstract algorithm with three key components: variation, selection, and retention (Dennett, 1995; Blackmore, 1999). This abstract algorithm constitutes a “universal Darwinism” that need not assume object permanence and can be profitably applied in many contexts beyond biological evolution. Thus, it is possible, without begging the question, to use formal models of evolution by natural selection to explore whether object permanence is an insight or not.

Jerry Fodor has criticized the theory of natural selection itself, arguing, for instance, that it impales itself with an intensional fallacy, viz., inferring from the premise that “evolution is a process in which creatures with adaptive traits are selected” to the conclusion that “evolution is a process in which creatures are selected for their adaptive traits” (Fodor and Piattelli-Palmarini, 2010). However, Fodor's critique seems wide of the mark (Futuyma, 2010) and the evidence for evolution by natural selection is overwhelming (Coyne, 2009; Dawkins, 2009).

What, then, do we find when we explore the evolution of perception using evolutionary games and genetic algorithms? The standard answer, at least among vision scientists, is that we should find that natural selection favors veridical perceptions, i.e., perceptions that accurately represent objective properties of the external world that exist when unperceived. Steven Palmer, for instance, in a standard graduate-level textbook, states that “Evolutionarily speaking, visual perception is useful only if it is reasonably accurate … Indeed, vision is useful precisely because it is so accurate. By and large, *what you see is what you get*. When this is true, we have what is called **veridical perception** … perception that is consistent with the actual state of affairs in the environment. This is almost always the case with vision … ” (Palmer, 1999).

The argument, roughly, is that those of our predecessors whose perceptions were more veridical had a competitive advantage over those whose perceptions were less veridical. Thus, the genes that coded for more veridical perceptions were more likely to propagate to the next generation. We are, with good probability, the offspring of those who, in each succeeding generation, perceived more truly, and thus we can be confident that our own perceptions are, in the normal case, veridical.

The conclusion that natural selection favors veridical perceptions is central to current Bayesian models of perception, in which perceptual systems use Bayesian inference to estimate true properties of the objective world, properties such as shape, position, motion, and reflectance (Knill and Richards, 1996; Geisler and Diehl, 2003). Objects exist and have these properties when unperceived, and the function of perception is to accurately estimate pre-existing properties.

However, when we actually study the evolution of perception using Monte Carlo simulations of evolutionary games and genetic algorithms, we find that natural selection does not, in general, favor perceptions that are true reports of objective properties of the environment. Instead, it generally favors perceptual strategies that are tuned to fitness (Mark et al., 2010; Hoffman et al., 2013; Marion, 2013; Mark, 2013).

Why? Several principles emerge from the simulations. First, there is no free information. For every bit of information one obtains about the external world, one must pay a price in energy, e.g., in calories expended to obtain, process and retain that information. And for every calorie expended in perception, one must go out and kill something and eat it to get that calorie. So natural selection tends to favor perceptual systems that, *ceteris paribus*, use fewer calories. One way to use fewer calories is to see less truth, especially truth that is not informative about fitness.

Second, for every bit of information one obtains about the external world, one must pay a price in time. More information requires, in general, more time to obtain and process. But in the real world where predators are on the prowl and prey must be wary, the race is often to the swift. It is the slower gazelle that becomes lunch for the swifter cheetah. So natural selection tends to favor perceptual systems that, *ceteris paribus*, take less time. One way to take less time is, again, to see less truth, especially truth that is not informative about fitness.

Third, in a world where organisms are adapted to niches and require homeostatic mechanisms, the fitness functions guiding their evolution are generally not monotonic functions of structures or quantities in the world. Too much salt or too little can be devastating; something in between is just right for fitness. The same goldilocks principle can hold for water, altitude, humidity, and so on. In these cases, perceptions that are tuned to fitness are *ipso facto* not tuned to the true structure of the world, because the two are not monotonically related; knowing the truth is not just irrelevant, it can be inimical, to fitness.

Fourth, in the generic case where noise and uncertainty are endemic to the perceptual process, a strategy that estimates a true state of the world and then uses the utility associated to that state to govern its decisions must throw away valuable information about utility. It will in general be driven to extinction by a strategy that does not estimate the true state of the world, and instead uses all the information about utility (Marion, 2013).

Fifth, more complex perceptual systems are more difficult to evolve. Monte Carlo simulations of genetic algorithms show that there is a combinatorial explosion in the complexity of the search required to evolve more complex perceptual systems. This combinatorial explosion itself is a selection pressure toward simpler perceptual systems.

In short, natural selection does not favor perceptual systems that see the truth in whole or in part. Instead, it favors perceptions that are fast, cheap, and tailored to guide behaviors needed to survive and reproduce. Perception is not about truth, it's about having kids. Genes coding for perceptual systems that increase the probability of having kids are *ipso facto* the genes that are more likely to code for perceptual systems in the next generation.

## The interface theory of perception

Natural selection favors perceptions that are useful though not true. This might seem counterintuitive, even to experts in perception. Palmer, for instance, in the quote above, makes the plausible claim that “vision is useful precisely because it is so accurate” (Palmer, 1999). Geisler and Diehl agree, taking it as obvious that “In general, (perceptual) estimates that are nearer the truth have greater utility than those that are wide of the mark” (Geisler and Diehl, 2002). Feldman also takes it as obvious that “it is clearly desirable (say from an evolutionary point of view) for an organism to achieve veridical percepts of the world” (Feldman, 2013). Knill and Richards concur that vision “… involves the evolution of an organism's visual system to match the structure of the world … ” (Knill and Richards, 1996).

This assumption that perceptions are useful to the extent that they are true is *prima facie* plausible, and it comports well with the assumption of object permanence. For if our perceptions report to us a three-dimensional world containing objects with specific shapes and positions, and if these perceptual reports have been shaped by evolution to be true, then we can be confident that those objects really do, in the normal case, exist and have their positions and shapes even when unperceived.

So we find it plausible that perceptions are useful only if true, and we find it deeply counterintuitive to think otherwise. But studies with evolutionary games and genetic algorithms flatly contradict this deeply held assumption. Clearly our intuitions need a little help here. How can we try to understand perceptions that are useful but not true?

Fortunately, developments in computer technology have provided a convenient and helpful metaphor: the desktop of a windows interface (Hoffman, 1998, 2009, 2011, 2012, 2013; Mausfeld, 2002; Koenderink, 2011a; Hoffman and Singh, 2012; Singh and Hoffman, 2013). Suppose you are editing a text file and that the icon for that file is a blue rectangle sitting in the lower left corner of the desktop. If you click on that icon you can open the file and revise its text. If you drag that icon to the trash, you can delete the file. If you drag it to the icon for an external hard drive, you can create a backup of the file. So the icon is quite useful.

But is it *true*? Well, the only visible properties of the icon are its position, shape, and color. Do these properties of the icon resemble the true properties of the file? Clearly not. The file is not blue or rectangular, and it's probably not in the lower left corner of the computer. Indeed, files don't have a color or shape, and needn't have a well-defined position (e.g., the bits of the file could be spread widely over memory). So to even ask if the properties of the icon are true is to make a category error, and to completely misunderstand the purpose of the interface. One can reasonably ask whether the icon is usefully related to the file, but not whether it truly resembles the file.

Indeed, a critical function of the interface is to *hide* the truth. Most computer users don't want to see the complexity of the integrated circuits, voltages, and magnetic fields that are busy behind the scenes when they edit a file. If they had to deal with that complexity, they might never finish their work on the file. So the interface is designed to allow the user to interact effectively with the computer while remaining largely ignorant of its true architecture.

Ignorant, also, of its true causal structure. When a user drags a file icon to an icon of an external drive, it looks obvious that the movement of the file icon to the drive icon *causes* the file to be copied. But this is just a useful fiction. The movement of the file icon causes nothing in the computer. It simply serves to guide the user's operation of a mouse, triggering a complex chain of causal events inside the computer, completely hidden from the user. Forcing the user to see the true causal chain would be an impediment, not a help.

Turning now to apply the interface metaphor to human perception, the idea is that natural selection has not shaped our perceptions to be insights into the true structure and causal nature of objective reality, but has instead shaped our perceptions to be a species-specific user interface, fashioned to guide the behaviors that we need to survive and reproduce. Space and time are the desktop of our perceptual interface, and three-dimensional objects are icons on that desktop.

Our interface gives the impression that it reveals true cause and effect relations. When one billiard ball hits a second, it certainly looks as though the first causes the second to careen away. But this appearance of cause and effect is simply a useful fiction, just as it is for the icons on the computer desktop.

There is an obvious rejoinder: “If that cobra is just an icon of your interface with no causal powers, why don't you grab it by the tail?” The answer is straightforward: “I don't grab the cobra for the same reason I don't carelessly drag my file icon to the trash—I could lose a lot of work. I don't take my icons *literally*: The file, unlike its icon, is not literally blue or rectangular. But I do take my icons *seriously*.”

Similarly, evolution has shaped us with a species-specific interface whose icons we must take seriously. If there is a cliff, don't step over. If there is a cobra, don't grab its tail. Natural selection has endowed us with perceptions that function to guide adaptive behaviors, and we ignore them at our own peril.

But, given that we must take our perceptions seriously, it does not follow that we must take them literally. Such an inference is natural, in the sense that most of us, even the brightest, make it automatically. When Samuel Johnson heard Berkeley's theory that “To be is to be perceived” he kicked a stone and said, “I refute it *thus*!” (Boswell, 1986) Johnson observed that one must take the stone seriously or risk injury. From this Johnson concluded that one must take the stone literally. But this inference is fallacious.

One might object that there still is an important sense in which our perceptual icon of, say, a cobra does resemble the true objective reality: The consequences for an observer of grabbing the tail of the cobra are precisely the consequences that would obtain if the objective reality were in fact a cobra. Perceptions and internal information-bearing structures are useful for fitness-preserving or enhancing behavior because there is some mutual information between the predicted utility of a behavior (like escaping) and its actual utility. If there's no mutual information and no mechanism for increasing mutual information, fitness is low and stays that way. Here we use mutual information in the sense of standard information theory (Cover and Thomas, 2006).

This point is well-taken. Our perceptual icons do give us genuine information about fitness, and fitness can be considered an aspect of objective reality. Indeed, in Gibson's ecological theory of perception, our perceptions primarily resonate to “affordances,” those aspects of the objective world that have important consequences for fitness (Gibson, 1979). While we disagree with Gibon's direct realism and denial of information processing in perception, we agree with his emphasis on the tuning of perception to fitness.

So we must clarify the relationship between truth and fitness. In evolutionary theory it is as follows. If *W* denotes the objective world then, for a fixed organism, state, and action, we can think of a fitness function to be a function *f*:*W* → [0,1], which assigns to each state *w* of *W* a fitness value *f*(*w*). If, for instance, the organism is a hungry cheetah and the action is eating, then *f* might assign a high fitness value to world state *w* in which fresh raw meat is available; but if the organism is a hungry cow then *f* might assign a low fitness value to the same state *w*.

If the true probabilities of states in the world are given by a probability measure *m* on *W*, then one can define a new probability measure *mf* on *W*, where for any event *A* of *W*, *mf*(*A*) is simply the integral of *f* over *A* with respect to *m*; *mf* must of course be normalized so that *mf*(*W*) = 1.

And here is the key point. A perceptual system that is tuned to maximize the mutual information with *m* will not, in general, maximize mutual information with *mf* (Cover and Thomas, 2006). Being tuned to truth, i.e., maximizing mutual information with *m*, is not the same as being tuned to fitness, i.e., maximizing mutual information with *mf*. Indeed, depending on the fitness function *f*, a perceptual system tuned to truth might carry little or no information about fitness, and vice versa. It is in this sense that the interface theory of perception claims that our perceptions are tuned to fitness rather than truth.

There is another rejoinder: “The interface metaphor is nothing new. Physicists have told us for more than a century that solid objects are really mostly empty space. So an apparently solid stone isn't the true reality, but its atoms and subatomic particles are.” Physicists have indeed said this since Rutherford published his theory of the atomic nucleus in 1911 (Rutherford, 1911). But the interface metaphor says something more radical. It says that space and time themselves are just a desktop, and that anything in space and time, including atoms and subatomic particles, are themselves simply icons. It's not just the moon that isn't there when one doesn't look, it's the atoms, leptons and quarks themselves that aren't there. Object permanence fails for microscopic objects just as it does for macroscopic.

This claim is, to contemporary sensibilities, radical. But there is a perspective on the intellectual evolution of humanity over the last few centuries for which the interface theory seems a natural next step. According to this perspective, humanity has gradually been letting go of the false belief that the way *H. sapiens* sees the world is an insight into objective reality.

Many ancient cultures, including the pre-Socratic Greeks, believed the world was flat, for the obvious reason that it looks that way. Aristotle became persuaded, on empirical grounds, that the earth is spherical, and this view gradually spread to other cultures. Reality, we learned, departed in important respects from some of our perceptions.

But then a geocentric model of the universe, in which the earth is at the center and everything revolves around it, still held sway. Why? Because that's the way things look to our unaided perceptions. The earth looks like it's not moving, and the sun, moon, planets, and stars look like they circle a stationary earth. Not until the work of Copernicus and Kepler did we recognize that once again reality differs, in important respects, from our perceptions. This was difficult to swallow. Galileo was forced to recant in the Vatican basement, and Giordano Bruno was burned at the stake. But we finally, and painfully, accepted the mismatch between our perceptions and certain aspects of reality.

The interface theory entails that these first two steps were mere warm up. The next step in the intellectual history of *H. sapiens* is a big one. We must recognize that *all* of our perceptions of space, time and objects no more reflect reality than does our perception of a flat earth. It's not just this or that aspect of our perceptions that must be corrected, it is *the entire framework* of a space-time containing objects, the fundamental organization of our perceptual systems, that must be recognized as a mere species-specific mode of perception rather than an insight into objective reality.

By this time it should be clear that, if the arguments given here are sound, then the current Bayesian models of object perception need more than tinkering around the edges, they need fundamental transformation. And this transformation will necessarily have ramifications for scientific questions well-beyond the confines of computational models of object perception.

One example is the mind-body problem. A theory in which objects and space-time do not exist unperceived and do not have causal powers, cannot propose that neurons—which by hypothesis do not exist unperceived and do not have causal powers—cause any of our behaviors or conscious experiences. This is so contrary to contemporary thought in this field that it is likely to be taken as a *reductio* of the view rather than as an alternative direction of inquiry for a field that has yet to construct a plausible theory.

## Definition of conscious agents

If our reasoning has been sound, then space-time and three-dimensional objects have no causal powers and do not exist unperceived. Therefore, we need a fundamentally new foundation from which to construct a theory of objects. Here we explore the possibility that consciousness is that new foundation, and seek a mathematically precise theory. The idea is that a theory of objects requires, first, a theory of subjects.

This is, of course, a non-trivial endeavor. Frank Wilczek, when discussing the interpretation of quantum theory, said, “The relevant literature is famously contentious and obscure. I believe it will remain so until someone constructs, within the formalism of quantum mechanics, an “observer,” that is, a model entity whose states correspond to a recognizable caricature of conscious awareness … That is a formidable project, extending well-beyond what is conventionally considered physics” (Wilczek, 2006).

The approach we take toward constructing a theory of consciousness is similar to the approach Alan Turing took toward constructing a theory of computation. Turing proposed a simple but rigorous formalism, now called the *Turing machine* (Turing, 1937; Herken, 1988). It consists of six components: (1) a finite set of states, (2) a finite set of symbols, (3) a special blank symbol, (4) a finite set of input symbols, (5) a start state, (6) a set of halt states, and (7) a finite set of simple transition rules (Hopcroft et al., 2006).

Turing and others then conjectured that a function is algorithmically computable if and only if it is computable by a Turing machine. This “Church-Turing Thesis” can't be proven, but it could in principle be falsified by a counterexample, e.g., by some example of a procedure that everyone agreed was computable but for which no Turing machine existed. No counterexample has yet been found, and the Church-Turing thesis is considered secure, even definitional.

Similarly, to construct a theory of consciousness we propose a simple but rigorous formalism called a *conscious agent*, consisting of six components. We then state the *conscious agent thesis*, which claims that every property of consciousness can be represented by some property of a conscious agent or system of interacting conscious agents. The hope is to start with a small and simple set of definitions and assumptions, and then to have a complete theory of consciousness arise as a series of theorems and proofs (or simulations, when complexity precludes proof). We want a theory of consciousness *qua consciousness*, i.e., of consciousness on its own terms, not as something derivative or emergent from a prior physical world.

No doubt this approach will strike many as *prima facie* absurd. It is a commonplace in cognitive neuroscience, for instance, that most of our mental processes are *unconscious* processes (Bargh and Morsella, 2008). The standard account holds that well more than 90% of mental processes proceed without conscious awareness. Therefore, the proposal that consciousness is fundamental is, to contemporary thought, an amusing anachronism not worth serious consideration.

This critique is apt. It's clear from many experiments that each of us is indeed unaware of most of the mental processes underlying our actions and conscious perceptions. But this is no surprise, given the interface theory of perception. Our perceptual interfaces have been shaped by natural selection to guide, quickly and cheaply, behaviors that are adaptive in our niche. They have not been shaped to provide exhaustive insights into truth. In consequence, our perceptions have endogenous limits to the range and complexity of their representations. It was not adaptive to be aware of most of our mental processing, just as it was not adaptive to be aware of how our kidneys filter blood.

We must be careful not to assume that limitations of our species-specific perceptions are insights into the true nature of reality. My friend's mind is not directly conscious to me, but that does not entail that my friend is unconscious. Similarly, most of my mental processes are not directly conscious to me, but that does not entail that they are unconscious. Our perceptual systems have finite capacity, and will therefore inevitably simplify and omit. We are well-advised not to mistake our omissions and simplifications for insights into reality.

There are of course many other critiques of an approach that takes consciousness to be fundamental: How can such an approach explain matter, the fundamental forces, the Big Bang, the genesis and structure of space-time, the laws of physics, evolution by natural selection, and the many neural correlates of consciousness? These are non-trivial challenges that must be faced by the theory of conscious agents. But for the moment we will postpone them and develop the theory of conscious agents itself.

*Conscious agent* is a technical term, with a precise mathematical definition that will be presented shortly. To understand the technical term, it can be helpful to have some intuitions that motivate the definition. The intuitions are just intuitions, and if they don't help they can be dropped. What does the heavy lifting is the definition itself.

A key intuition is that consciousness involves three processes: *perception*, *decision*, and *action*.

In the process of perception, a conscious agent interacts with the world and, in consequence, has conscious experiences.

In the process of decision, a conscious agent chooses what actions to take based on the conscious experiences it has.

In the process of action, the conscious agent interacts with the world in light of the decision it has taken, and affects the state of the world.

Another intuition is that we want to avoid unnecessarily restrictive assumptions in constructing a theory of consciousness. Our conscious visual experience of nearby space, for instance, is approximately Euclidean. But it would be an unnecessary restriction to require that *all* of our perceptual experiences be represented by Euclidean spaces.

However it does seem necessary to discuss the *probability* of having a conscious experience, of making a particular decision, and of making a particular change in the world through action. Thus, it seems necessary to assume that we can represent the world, our conscious experiences, and our possible actions with probability spaces.

We also want to avoid unnecessarily restrictive assumptions about the *processes* of perception, decision, and action. We might find, for instance, that a particular decision process maximizes expected utility, or minimizes expected risk, or builds an explicit model of the self. But it would be an unnecessary restriction to require this of all decisions.

However, when considering the processes of perception, decision and action, it does seem necessary to discuss *conditional probability*. It seems necessary, for instance, to discuss the conditional probability of deciding to take a specific action given a specific conscious experience, the conditional probability of a particular change in the world given that a specific action is taken, and the conditional probability of a specific conscious experience given a specific state of the world.

A general way to model such conditional probabilities is by the mathematical formalism of Markovian kernels (Revuz, 1984). One can think of a Markovian kernel as simply an indexed list of probability measures. In the case of perception, for instance, a Markovian kernel might specify that if the state of the world is *w*_1_, then here is a list of the probabilities for the various conscious experiences that might result, but if the state of the world is *w*_2_, then here is a different list of the probabilities for the various conscious experiences that might result, and so on for all the possible states of the world. A Markovian kernel on a finite set of states can be written as matrix in which the entries in each row sum to 1.

A Markovian kernel can also be thought of as an *information channel*. Cover and Thomas, for instance, define “a discrete channel to be a system consisting of an input alphabet *X* and output alphabet *Y* and a probability transition matrix *p*(*x*|*y*) that expresses the probability of observing the output symbol *y* given that we send the symbol *x*” (Cover and Thomas, 2006). Thus, a discrete channel is simply a Markovian kernel.

So, each time a conscious agent interacts with the world and, in consequence, has a conscious experience, we can think of this interaction as a message being passed from the world to the conscious agent over a channel. Similarly, each time the conscious agent has a conscious experience and, in consequence, decides on an action to take, we can think of this decision as a message being passed over a channel within the conscious agent itself. And when the conscious agent then takes the action and, in consequence, alters the state of the world, we can think of this as a message being passed from the conscious agent to the world over a channel. In the discrete case, we can keep track of the number of times each channel is used. That is, we can count the number of messages that are passed over each channel. Assuming that all three channels (perception, decision, action) all work in lock step, we can use one counter, *N*, to keep track of the number of messages that are passed.

**Figure 1:**
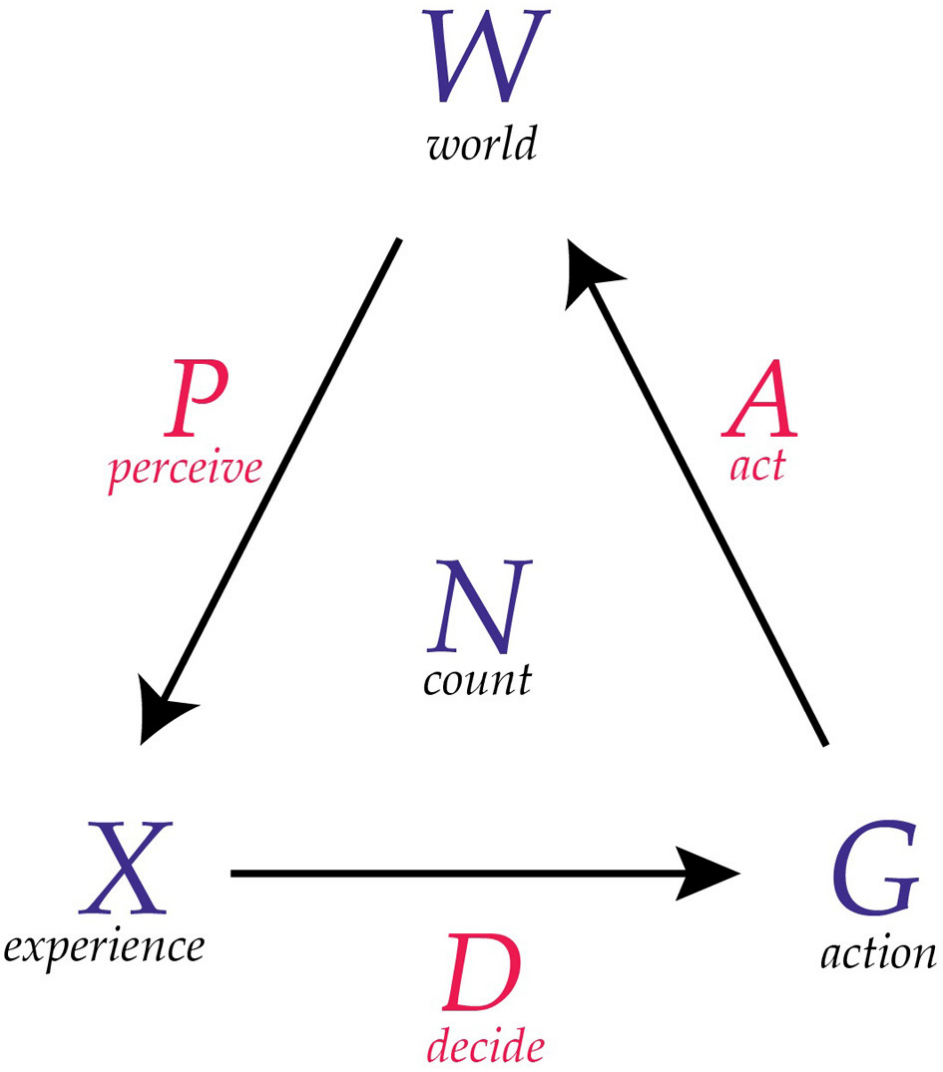
**A diagram of a conscious agent**. A conscious agent has six components as illustrated here. The maps *P*, *D*, and *A* can be thought of as communication channels.

These are some of the intuitions that underlie the definition of conscious agent that we will present. These intuitions can be represented pictorially in a diagram, as shown in Figure 1. The channel *P* transmits messages from the world *W*, leading to conscious experiences *X*. The channel *D* transmits messages from *X*, leading to actions *G*. The channel *A* transmits messages from *G* that are received as new states of *W*. The counter *N* is an integer that keeps track of the number of messages that are passed on each channel.

In what follows we will be using the notion of a measurable space. Recall that a measurable space, (*X*, **X**), is a set *X* together with a collection **X** of subsets of *X*, called *events*, that satisfies three properties: (1) *X* is in ***X***; (2) **X** is closed under complement (i.e., if a set *A* is in **X** then the complement of *A* is also in **X**); and (3) **X** is closed under countable union. The collection of events **X** is a σ-algebra (Athreya and Lahiri, 2006). A probability measure assigns a probability to each event in **X**.

With these intuitions, we now present the formal definition of a conscious agent where, for the moment, we simply assume that the world is a measurable space (*W*, **W**).

**Definition 1**. A *conscious agent*, *C*, is a six-tuple

(1) C=((X,X),(G,G),P,D,A,N)),

where:

1. (*X*, **X**) and (*G*, **G**) are measurable spaces;
2. *P*: *W* × **X** → [0, 1], *D*: *X* × **G** → [0, 1], *A*: *G* × **W** → [0, 1] are Markovian kernels; and
3. *N* is an integer.

For convenience we will often write a conscious agent *C* as

(2) C=(X,G,P,D,A,N),

omitting the σ-algebras.

Given that *P*, *D*, and *A* are channels, each has a *channel capacity*, viz., a highest rate of bits per channel use, at which information can be sent across the channel with arbitrarily low chance of error (Cover and Thomas, 2006).

The formal structure of a conscious agent, like that of a Turing machine, is simple. Nevertheless, we will propose, in the next section, a “conscious-agent thesis” which, like the Church-Turing thesis, claims wide application for the formalism.

## Conscious realism

One glaring feature of the definition of a conscious agent is that it involves the world, *W*. This is not an arbitrary choice; *W* is required to define the perceptual map *P* and action map *A* of the conscious agent.

This raises the question: What is the world? If we take it to be the space-time world of physics, then the formalism of conscious agents is dualistic, with some components (e.g., *X* and *G*) referring to consciousness and another, viz., *W*, referring to a physical world.

We want a non-dualistic theory. Indeed, the monism we want takes consciousness to be fundamental. The formalism of conscious agents provides a precise way to state this monism.

**Hypothesis 1***. Conscious realism*: The world *W* consists entirely of conscious agents.

Conscious realism is a precise hypothesis that, of course, might be precisely wrong. We can explore its theoretical implications in the normal scientific manner to see if they comport well with existing data and theories, and make predictions that are novel, interesting and testable.

### Two conscious agents

**Figure 2:**
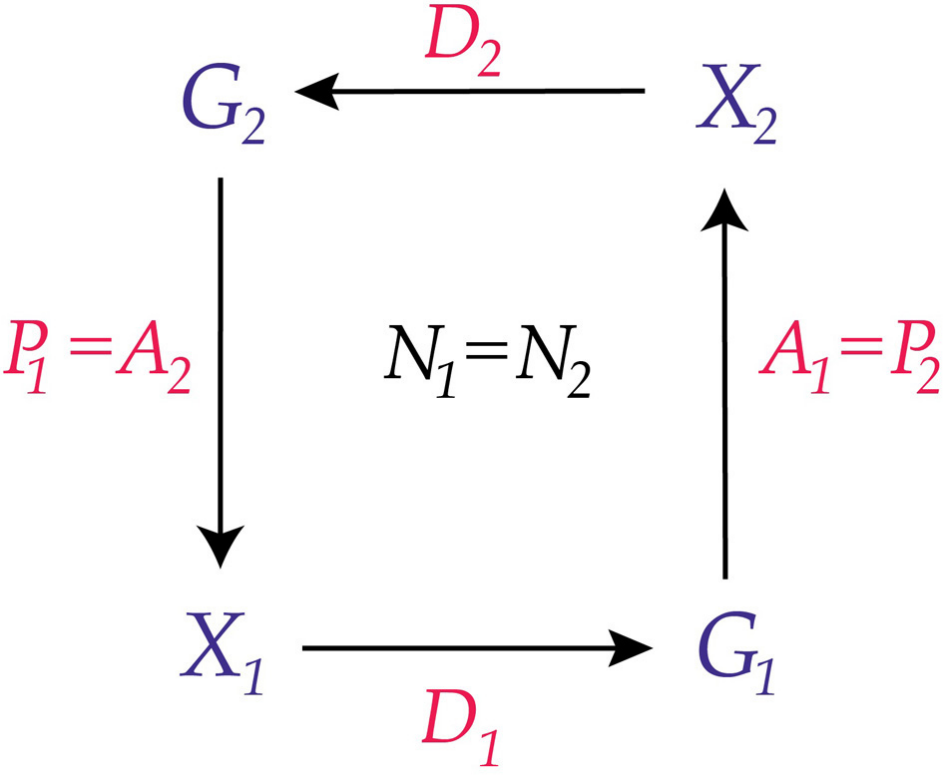
**Two conscious agents, *C*_1_ and *C*_2_**. Each is part of the world *W* for the other conscious agent. The lower part of the diagram represents *C*_1_ and the upper part represents *C*_2_. This creates an *undirected combination* of *C*_1_ and *C*_2_, a concept we define in section The Combination Problem.

Conscious realism can be expressed mathematically in a simple form. Consider the elementary case, in which the world *W* of one conscious agent,

(3) $$C_{1}=(X_{1},G_{1},P_{1},D_{1},A_{1},N_{1}),$$

contains just *C*_1_ and one other agent,

(4) $$C_{2}=(X_{2},G_{2},P_{2},D_{2},A_{2},N_{2}),$$

and vice versa. This is illustrated in Figure 2.

Observe that although *W* is the world it cannot properly be called, in this example, the *external* world of *C*_1_ or of *C*_2_ because *C*_1_ and *C*_2_ are each part of *W*. This construction of *W* requires the compatibility conditions

(5) $$P_{1}=A_{2},$$

(6) $$P_{2}=A_{1},$$

(7) $$N_{1}=N_{2}.$$

These conditions mean that the perceptions of one conscious agent are identical to the actions of the other, and that their counters are synchronized. To understand this, recall that we can think of *P*_1_, *P*_2_, *A*_1_, and *A*_2_ as *information channels*. So interpreted, conditions (5) and (6) state that the action channel of one agent is the same information channel as the perception channel of the other agent. Condition (7) states that the channels of both agents operate in synchrony.

**Figure 3:**
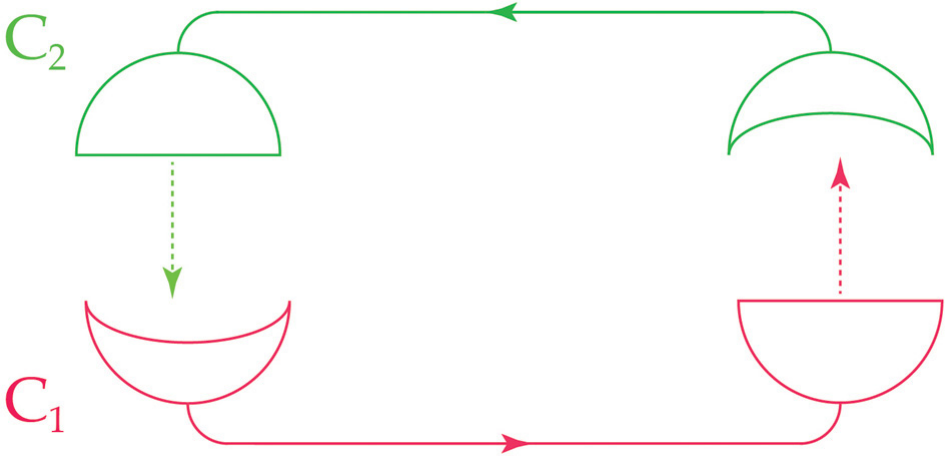
**Two adjacent conscious agents, *C*_1_ and *C*_2_**. Each agent receives messages from the other (indicated by the concave receivers) and sends messages to the other (indicated by the semicircular transmitters). Arrows show the direction of information flow.

If two conscious agents *C*_1_ and *C*_2_ satisfy the commuting diagram of Figure 2, then we say that they are *joined* or *adjacent*: the experiences and actions of *C*_1_ affect the probabilities of experiences and actions for *C*_2_ and vice versa. Figure 3 illustrates the ideas so far.

We can simplify the diagrams further and simply write *C*_1_—*C*_2_ to represent two adjacent conscious agents.

### Three conscious agents

Any number of conscious agents can be joined. Consider the case of three conscious agents,

(8) $$C_{i}=(X_{i},G_{i},P_{i},D_{i},A_{i},N_{i}),i=1,2,3.$$

**Figure 4:**
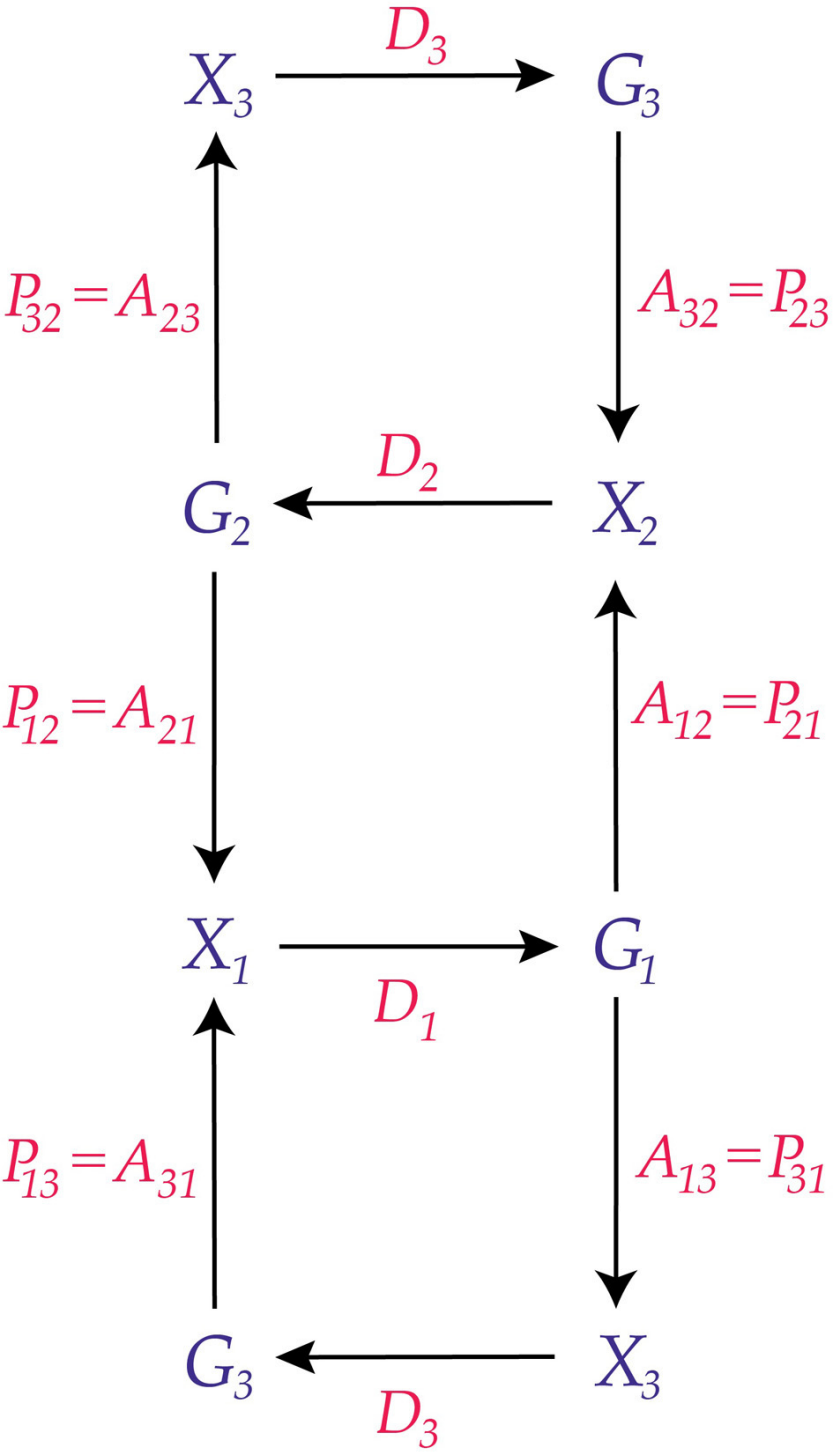
**Three adjacent conscious agents**. The third agent is replicated at the **top** and **bottom** of the diagram for visual simplicity.

**Figure 5:**
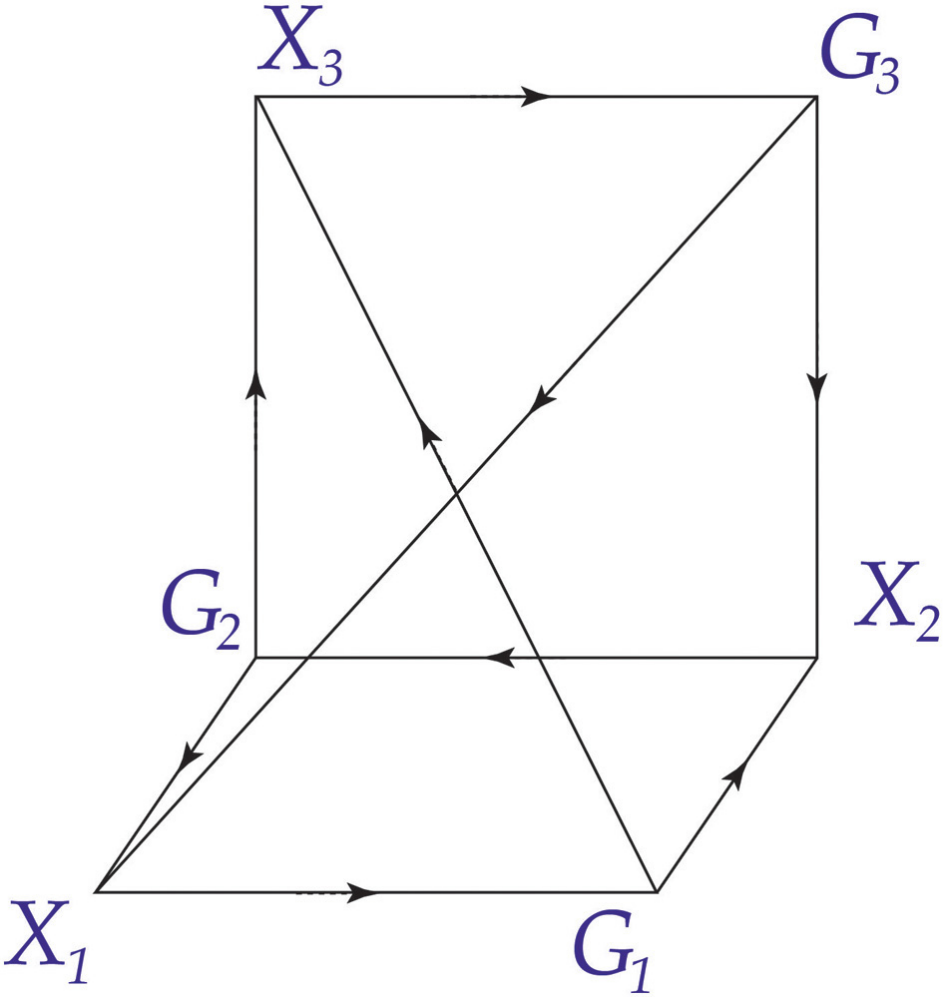
**Three adjacent conscious agents**. This is a compact representation of the diagram in Figure 4.

This is illustrated in Figure 4, and compactly in Figure 5.

Because *C*_1_ interacts with *C*_2_ and *C*_3_, its perceptions are affected by both *C*_2_ and *C*_3_. Thus, its perception kernel, *P*_1_, must reflect the inputs of *C*_2_ and *C*_3_. We write it as follows:

(9) $$P_{1}=P_{12}⊗P_{13}:(G_{2}\times G_{3})\times X_{1}\to [0,1],$$

where

(10) $$X_{1}=\sigma (X_{12}\times X_{13}),$$

(*X*_12_, **X**_12_) is the measurable space of perceptions that *C*_1_ can receive from *C*_2_, and (*X*_13_, **X**_13_) is the measurable space of perceptions that *C*_1_ can receive from *C*_3_, and **σ**(**X**_12_ × **X**_13_) denotes the σ-algebra generated by the Cartesian product of **X**_12_ and **X**_13_. The tensor product *P*_1_ of (9) is given by the formula

(11) $$P_{1}\left(\left(g_{2},g_{3}),(x_{12},x_{13}\right)\right)=P_{12}(g_{2},x_{12})P_{13}(g_{3},x_{13}),$$

where *g*_2_ ∈ *G*_2_, *g*_3_ ∈ *G*_3_, *x*_12_ ∈ **X**_12_, and *x*_13_ ∈ **X**_13_. Note that (11) allows that the perceptions that *C*_1_ gets from *C*_2_ could be entirely different from those it gets from *C*_3_, and expresses the probabilistic independence of these perceptual inputs. In general, *X*_**12**_ need not be identical to *X*_**13**_, since the kinds of perceptions that *C*_1_ can receive from *C*_2_ need not be the same as the kinds of perceptions that *C*_1_ can receive from *C*_3_.

Because *C*_1_ interacts with *C*_2_ and *C*_3_, its actions affect both. However, the way *C*_1_ acts on *C*_2_ might differ from how it acts on *C*_3_, and the definition of its action kernel, *A*_1_, must allow for this difference of action. Therefore, we define the action kernel, *A*_1_, to be the tensor product

(12) $$A_{1}=A_{12}⊗A_{13}:G_{1}\times \sigma (X_{2}\times X_{3})\to [0,1],$$

where

(13) $$G_{1}=G_{12}\times G_{13},$$

(*G*_12_, **G**_12_) is the measurable space of actions that *C*_1_ can take on *C*_2_, and (*G*_13_, **G**_13_) is the measurable space of actions that *C*_1_ can take on *C*_3_.

**Figure 6:**
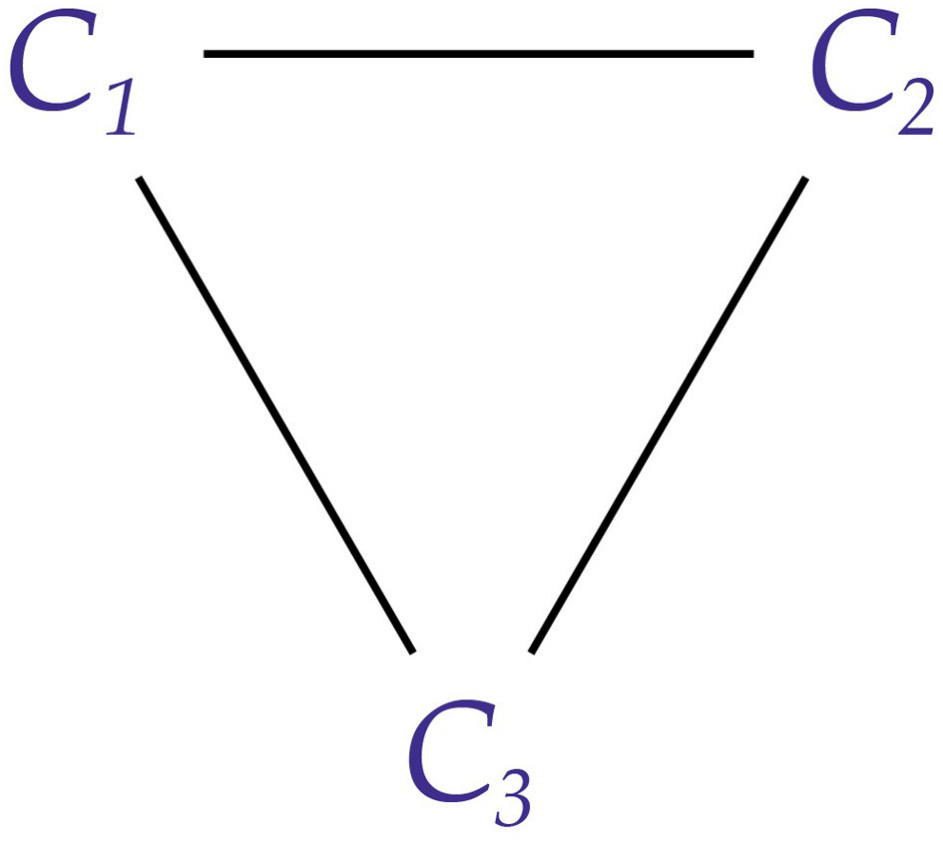
**Three conscious agents whose graph is complete**.

In this situation, the three conscious agents have the property that every pair is adjacent; we say that the graph of the three agents is *complete*. This is illustrated in Figure 6.

**Figure 7:**
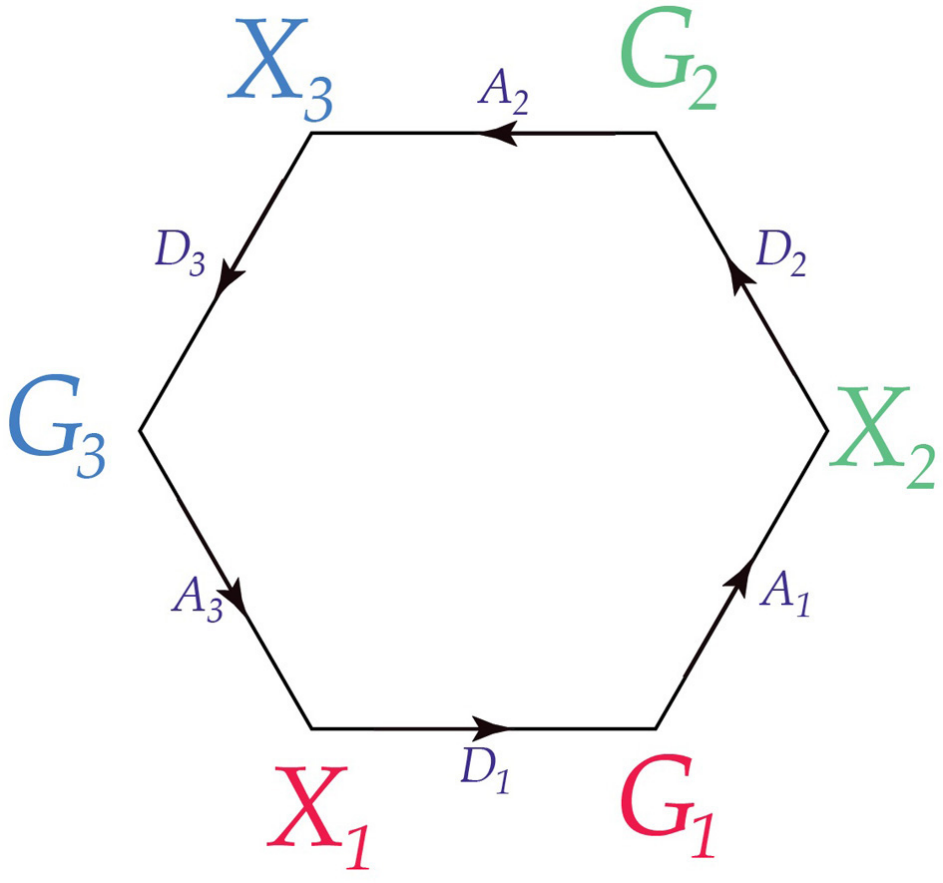
**Three conscious agents with directed joins**. Here we assume *A*_1_ = *P*_2_, *A*_2_ = *P*_3_, and *A*_3_ = *P*_1_.

So far we have considered joins that are undirected, in the sense that if *C*_1_ sends a message to *C*_2_ then *C*_2_ sends a message to *C*_1_. However, it is also possible for conscious agents to have *directed joins*. This is illustrated in Figure 7. In this case, *C*_1_ sends a message to *C*_2_ and receives a message from *C*_3_, but receives no message from *C*_2_ and sends no message to *C*_3_. Similar remarks hold, *mutatis mutandis*, for *C*_2_ and *C*_3_.

**Figure 8:**
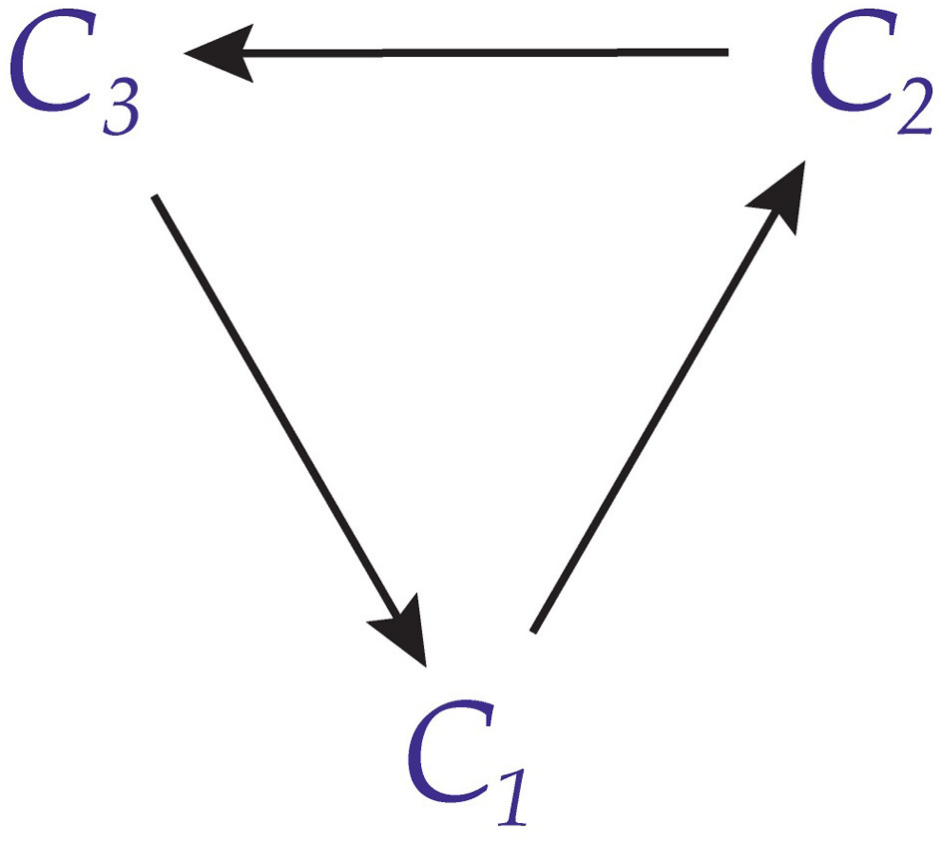
**Simplified graph of three conscious agents with directed joins**.

Figure 7 can be simplified as shown in Figure 8.

Directed joins can model the standard situation in visual perception, in which there are multiple levels of visual representations, one level building on others below it. For instance, at one level there could be the construction of 2D motions based on a solution to the correspondence problem; at the next level there could be a computation of 3D structure from motion, based on the 2D motions computed at the earlier level (Marr, 1982). So an agent *C*_1_ might solve the correspondence problem and pass its solution to *C*_2_, which solves the structure-from-motion problem, and then passes its solution to *C*_3_, which does object recognition.

We can join any number of conscious agents into any multi-graph, where nodes denote agents and edges denote directed or undirected joins between agents (Chartrand and Ping, 2012). The nodes can have any finite degree, i.e., any finite number of edges. As a special case, conscious agents can be joined to form deterministic or non-deterministic cellular automata (Ceccherini-Silberstein and Coornaert, 2010) and universal Turing machines (Cook, 2004).

## Dynamics of two conscious agents

Two conscious agents

(14) $$C_{1}=(X_{1},G_{1},P_{1},D_{1},A_{1},N_{1}),$$

and

(15) $$C_{2}=(X_{2},G_{2},P_{2},D_{2,}A_{2},N_{2}),$$

can be joined, as illustrated in Figure 2, to form a dynamical system. Here we discuss basic properties of this dynamics.

The state space, *E*, of the dynamics is *E* = *X*_1_ × *G*_1_ × *X*_2_ × *G*_2_, with product σ-algebra **E**. The idea is that for the current step, *t* ∈ *N*, of the dynamics, the state can be described by the vector (*x*_1_(*t*), *g*_1_(*t*), *x*_2_(*t*), *g*_2_(*t*)), and based on this state four actions happen simultaneously: (1) agent *C*_1_ experiences the perception *x*_1_(*t*) ∈ *X*_1_ and decides, according to *D*_1_, on a specific action *g*_1_(*t*) ∈ *G*_1_ to take at step *t* + 1; (2) agent *C*_1_, using *A*_1_, takes the action *g*_1_(*t*) ∈ *G*_1_; (3) agent *C*_2_ experiences the perception *x*_2_(*t*) ∈ *X*_2_ and decides, according to *D*_2_, on a specific action *g*_2_(*t*) ∈ *G*_2_ to take at step *t* + 1; (4) agent *C*_2_, using *A*_2_, takes the action *g*_2_(*t*) ∈ *G*_2_.

Thus, the state evolves by a kernel

(16) L:E×E→[0,1],

which is given, for state *e* = (*x*_1_(*t*), *g*_1_(*t*), *x*_2_(*t*), *g*_2_(*t*)) ∈ at time *t* and event *B* ∈ **E**, comprised of a measurable set of states of the form (*x*_1_(*t* + 1), *g*_1_(*t* + 1), *x*_2_(*t* + 1), *g*_2_(*t* + 1)), by

(17) $$\begin{array}{l} L(e,B)=\int_{B}A_{2}(g_{2}(t),dx_{1}(t+1))D_{1}(x_{1}(t),dg_{1}(t+1))A_{1}(g_{1}(t),\\ \;dx_{2}(t+1))D_{2}(x_{2}(t),dg_{2}(t+1)). \end{array}$$

This is not kernel composition; it is simply multiplication of the four kernel values. The idea is that at each step of the dynamics each of the four kernels acts simultaneously and independently of the others to transition the state (*x*_1_(*t*), *g*_1_(*t*), *x*_2_(*t*), *g*_2_(*t*)) to the next state (*dx*_1_(*t* + 1), *dg*_1_(*t* + 1), *dx*_2_(*t* + 1), *dg*_2_(*t* + 1)).

### First example of asymptotic behavior

For concreteness, consider the simplest possible case where (1) *X*_1_, *G*_1_, *X*_2_, and *G*_2_ each have only two states which, using Dirac notation, we denote |0〉 and |1〉, and (2) each of the kernels *A*_2_, *D*_1_, *A*_1_, and *D*_2_ is a 2 × 2 identity matrix.

There are total of 2^4^ = 16 possible states for the dynamics of the two agents, which we can write as |0000〉, |0001〉, |0010〉, … |1111〉, where the leftmost digit is the state of *X*_1_, the next digit the state of *G*_1_, the next of *X*_2_, and the rightmost of *G*_2_.

The asymptotic (i.e., long-term) dynamics of these two conscious agents can be characterized by its absorbing sets and their periods. Recall that an absorbing set for such a dynamics is a smallest set of states that acts like a roach motel: once the dynamics enters the absorbing set it never leaves, and it forever cycles periodically through the states within that absorbing set. It is straightforward to verify that for the simple dynamics of conscious agents just described, the asymptotic behavior is as follows:

1. {|0000〉} is absorbing with period 1;
2. {|1111〉} is absorbing with period 1;
3. {|0101〉, |1010〉} is absorbing with period 2;
4. {|0001〉, |1000〉, |0100〉, |0010〉} is absorbing with period 4, and cycles in that order;
5. {|0011〉, |1001〉, |1100〉, |0110〉} is absorbing with period 4, and cycles in that order;
6. {|0111〉, |1011〉, |1101〉, |1110〉} is absorbing with period 4, and cycles in that order.

### Second example of asymptotic behavior

If we alter this dynamics by simply changing the kernel *D*_1_ from an identity matrix to the matrix *D*_1_ = ((0,1),(1,0)), then the asymptotic behavior changes to the following:

1. {|0000〉, |0100〉, |0110〉, |0111〉, |1111〉, |1011〉, |1001〉, |1000〉} is absorbing with period 8, and cycles in that order;
2. {|0001〉, |1100〉, |0010〉, |0101〉, |1110〉, |0011〉, |1101〉, |1010〉} is absorbing with period 8, and cycles in that order.

If instead of changing *D*_1_ we changed *D*_2_ (or *A*_1_ or *A*_2_) to ((0,1),(1,0)), we would get the same asymptotic behavior. Thus, in general, an asymptotic behavior corresponds to an equivalence class of interacting conscious agents.

The range of possible dynamics of pairs of conscious agents is huge, and grows as one increases the richness of the state space *E* and, therefore, the set of possible kernels. The possibilities increase as one considers dynamical systems of three or more conscious agents, with all the possible directed and undirected joins among them, forming countless connected multi-graphs or amenable groups.

With this brief introduction to the dynamics of conscious agents we are now in a position to state another key hypothesis.

**Hypothesis 2**. *Conscious-agent thesis*. Every property of consciousness can be represented by some property of a dynamical system of conscious agents.

## The combination problem

Conscious realism and the conscious-agent thesis are strong claims, and face a tough challenge: Any theory that claims consciousness is fundamental must solve the *combination problem* (Seager, 1995; Goff, 2009; Blamauer, 2011; Coleman, 2014). William Seager describes this as “the problem of explaining how the myriad elements of ‘atomic consciousness’ can be combined into a new, complex and rich consciousness such as that we possess” (Seager, 1995).

William James saw the problem back in 1890: “Where the elemental units are supposed to be feelings, the case is in no wise altered. Take a hundred of them, shuffle them and pack them as close together as you can (whatever that may mean); still each remains the same feeling it always was, shut in its own skin, windowless, ignorant of what the other feelings are and mean. There would be a hundred-and-first feeling there, if, when a group or series of such feelings were set up, a consciousness belonging to the group as such should emerge. And this 101st feeling would be a totally new fact; the 100 original feelings might, by a curious physical law, be a signal for its creation, when they came together; but they would have no substantial identity with it, nor it with them, and one could never deduce the one from the others, or (in any intelligible sense) say that they evolved it. … The private minds do not agglomerate into a higher compound mind” (James, 1890/2007).

There are really two combination problems. The first is the combination of phenomenal *experiences*, i.e., of qualia. For instance, one's taste experiences of salt, garlic, onion, basil and tomato are somehow combined into the novel taste experience of a delicious pasta sauce. What is the relationship between one's experiences of the ingredients and one's experience of the sauce?

The second problem is the combination of *subjects* of experiences. In the sauce example, a single subject experiences the ingredients and the sauce, so the problem is to combine experiences within a single subject. But how can we combine subjects themselves to create a new unified subject? Each subject has its point of view. How can different points of view be combined to give a new, single, point of view?

No rigorous theory has been given for combining phenomenal experiences, but there is hope. Sam Coleman, for instance, is optimistic but notes that “there will have to be some sort of qualitative blending or pooling among the qualities carried by each ultimate: if each ultimate's quality showed up as such in the macro-experience, it would lack the notable homogeneity of (e.g., color experience, and plausibly some mixing of basic qualities is required to obtain the qualities of macro-experience” (Coleman, 2014).

Likewise, no rigorous theory has been given for combining subjects. But here there is little hope. Thomas Nagel, for instance, says “Presumably the components out of which a point of view is constructed would not themselves have to have points of view” (Nagel, 1979). Coleman goes further, saying, “it is impossible to explain the generation of a macro-subject (like one of us) in terms of the assembly of micro-subjects, for, as I show, subjects cannot combine” (Coleman, 2014).

So at present there is the hopeful, but unsolved, problem of combining experiences and the hopeless problem of combining subjects.

The theory of conscious agents provides two ways to combine conscious agents: *undirected combinations* and *directed combinations*. We prove this, and then consider the implications for solving the problems of combining experiences and combining subjects.

**Theorem 1**. (*Undirected Join Theorem*.) An undirected join of two conscious agents creates a new conscious agent.

***Proof***. (*By construction*.) Let two conscious agents

(18) $$C_{1}=((X_{1},X_{1}),(G_{1},G_{1}),P_{1},D_{1},A_{1},N_{1}),$$

and

(19) $$C_{2}=((X_{2},X_{2}),(G_{2},G_{2}),P_{2},D_{2},A_{2},N_{2}),$$

have an undirected join. Let

(20) C=((X,X),(G,G),P,D,A,N))

where

(21) $$X=X_{1}\times X_{2},$$

(22) $$G=G_{1}\times G_{2},$$

(23) $$P=P_{1}⊗P_{2}:G^{T}\times X\to [0,1],$$

(24) $$D\;=D_{1}⊗D_{2}:X\times G\to [0,1],$$

(25) $$A\;=A_{1}⊗A_{2}:G\times X^{T}\to [0,1],$$

(26) $$N\text{ = }N_{\text{1}}\text{ = }N_{\text{2}}\text{,}$$

where superscript *T* indicates transpose, e.g., *X^T^* = X_2_ × X_1_; where **X** is the σ-algebra generated by the Cartesian product of **X**_1_ and **X**_2_; where **G** is the σ-algebra generated by **G**_1_ and **G**_2_; and where the Markovian kernels *P*, *D*, and *A* are given explicitly, in the discrete case, by

(27) $$\begin{array}{lll} P((g_{2},g_{1}),(x_{1},x_{2}))\; & = & P_{1}⊗P_{2}((g_{2},g_{1}),(x_{1},x_{2}))\\ & & =P_{1}(g_{2},x_{1})P_{2}(g_{1},x_{2}), \end{array}$$

(28) $$\begin{array}{lll} D((x_{1},x_{2}),(g_{1},g_{2}))\; & = & D_{1}⊗D_{2}((x_{1},x_{2}),(g_{1},g_{2}))\\ & & =D_{1}(x_{1},g_{1})D_{2}(g_{1},g_{2}), \end{array}$$

(29) $$\begin{array}{lll} A((g_{1},g_{2}),(x_{2},x_{1})) & = & A_{1}⊗A_{2}((g_{1},g_{2}),(x_{2},x_{1}))\\ & & =A_{1}(g_{1},x_{2})A_{2}(g_{2},x_{1}), \end{array}$$

where *g*_1_ ∈ *G*_1_, *g*_2_ ∈ *G*_2_, *x*_1_ ∈ *X*_1_, and *x*_2_ ∈ *X*_2_. Then *C* satisfies the definition of a conscious agent.       □

Thus, the undirected join of two conscious agents (illustrated in Figure 2) creates a single new conscious agent that we call their *undirected combination*. It is straightforward to extend the construction in Theorem 1 to the case in which more than two conscious agents have an undirected join. In this case the joined agents create a single new agent that is their undirected combination.

**Theorem 2**. (*Directed Join Theorem*.) A directed join of two conscious agents creates a new conscious agent.

***Proof***. (*By construction*.) Let two conscious agents

(30) $$C_{1}=((X_{1},X_{1}),(G_{1},G_{1}),P_{1},D_{1},A_{1},N_{1}),$$

and

(31) $$C_{2}=((X_{2},X_{2}),(G_{2},G_{2}),P_{2},D_{2},A_{2},N_{2}),$$

have the directed join *C*_1_ → C_2_. Let

(32) C=((X,X),(G,G),P,D,A,N))

where

(33) $$X\text{ = }X_{\text{1}},$$

(34) $$G\text{ = }G_{\text{2}}$$

(35) $$P\text{ = }P_{\text{1}}$$

(36) $$D\;=D_{1}A_{1}D_{2}:X_{1}\times G_{2}\to [0,1],$$

(37) $$A\text{ = }A_{\text{2}}$$

(38) $$N\text{ = }N_{\text{1}}\text{ = }N_{\text{2}}$$

where *D*_1_*A*_1_*D*_2_ denotes kernel composition. Then *C* satisfies the definition of a conscious agent.       □

Thus, the directed join of two conscious agents creates a single new conscious agent that we call their *directed combination*. It is straightforward to extend the construction in Theorem 2 to the case in which more than one conscious agent has a directed join to *C*_2_. In this case, all such agents, together with *C*_2_, create a new agent that is their directed combination.

Given Theorems 1 and 2, we make the following

**Conjecture 3**: (*Combination Conjecture*.) Given any pseudograph of conscious agents, with any mix of directed and undirected edges, then any subset of conscious agents from the pseudograph, adjacent to each other or not, can be combined to create a new conscious agent.

How do these theorems address the problems of combining experiences and subjects? We consider first the combination of experiences.

Suppose *C*_1_ has a space of possible perceptual experiences *X*_1_, and *C*_2_ has a space of possible perceptual experiences *X*_2_. Then their undirected join creates a new conscious agent *C* that has a space of possible perceptual experiences *X* = *X*_1_ × *X*_2_. In this case, *C* has possible experiences that are not possible for *C*_1_ or *C*_2_. If, for instance, *C*_1_ can see only achromatic brightness, and *C*_2_ can see only variations in hue, then *C* can see hues of varying brightness. Although *C*'s possible experiences *X* are the Cartesian product of *X*_1_ and *X*_2_, nevertheless *C* might exhibit perceptual dependence between *X*_1_ and *X*_2_, due to feedback inherent in an undirected join (Maddox and Ashby, 1996; Ashby, 2000).

For a directed join *C*_1_ → C_2_, the directed-combination agent C has a space of possible perceptual experiences *X* = *X*_1_. This might suggest that no combination of experiences takes place. However, *C* has a decision kernel *D* that is given by the kernel product *D*_1_*A*_1_*D*_2_. This product integrates (in the literal sense of integral calculus) over the entire space of perceptual experiences *X*_2_, making these perceptual experiences an integral part of the decision process. This comports well with evidence that there is something it is like to make a decision (Nahmias et al., 2004; Bayne and Levy, 2006), and suggests the intriguing possibility that the phenomenology of decision making is intimately connected with the spaces of perceptual experiences that are integrated in the decision process. This is an interesting prediction of the formalism of conscious agents, and suggests that solution of the combination problem for experience will necessarily involve the integration of experience with decision-making.

We turn now to the combination of subjects. Coleman describes subjects as follows: “The idea of being a subject goes with being an experiential entity, something conscious of phenomenal qualities. That a given subject has a particular phenomenological point of view can be taken as saying that there exists a discrete ‘sphere’ of conscious-experiential goings-on corresponding to this subject, with regard to which other subjects are distinct in respect of the phenomenal qualities they experience, and they have no direct (i.e., experiential) access to the qualitative field enjoyed by the first subject. A subject, then, can be thought of as a point of view annexed to a private qualitative field” (Coleman, 2014).

A conscious agent *C*_i_ is a subject in the sense described by Coleman. It has a distinct sphere, *X_i_*, of “conscious-experiential goings-on” and has no direct experiential access to the sphere, *X_j_*, of experiences of any other conscious agent *C*_j_. Moreover, a conscious agent is a subject in the further sense of being an *agent*, i.e., making decisions and taking actions on its own. Thus, according to the theory being explored here a subject, a point of view, is a six-tuple that satisfies the definition of a conscious agent.

The problem with combining subjects is, according to Goff, that “It is never the case that the existence of a number (one or more) of subjects of experience with certain phenomenal characters a priori entails the existence of some other subject of experience” (Goff, 2009).

Coleman goes further, saying that “The combination of subjects is a demonstrably incoherent notion, not just one lacking in a priori intelligibility … ” (Coleman, 2014). He explains why: “… a set of points of view have nothing to contribute as such to a single, unified successor point of view. Their essential property defines them against it: in so far as they are points of view they are experientially distinct and isolated—they have different streams of consciousness. The diversity of the subject-set, of course, derives from the essential oneness of any given member: since each subject is essentially a oneness, a set of subjects are essentially diverse, for they must be a set of onenesses. Essential unity from essential diversity … is thus a case of emergence … ”

The theory of conscious agents proposes that a subject, a point of view, is a six-tuple that satisfies the definition of conscious agent. The directed and undirected join theorems give constructive proofs of how conscious agents and, therefore, points of view, can be combined to create a new conscious agent, and thus a new point of view. The original agents, the original subjects, are not destroyed in the creation of the new agent, the new subject. Instead the original subjects structurally contribute in an understandable, indeed mathematically definable, fashion to the structure and properties of the new agent. The original agents are, indeed, influenced in the process, because they interact with each other. But they retain their identities. And the new agent has new properties not enjoyed by the constituent agents, but which are intelligible from the structure and interactions of the constituent agents. In the case of undirected combination, for instance, we have seen that the new agent can have periodic asymptotic properties that are not possessed by the constituent agents but that are intelligible—and thus not emergent in a brute sense—from the structures and interactions of the constituent agents.

Thus, in short, the theory of conscious agents provides the first rigorous theoretical account of the combination of subjects. The formalism is rich with deductive implications to be explored. The discussion here is just a start. But one hint is the following. The undirected combination of two conscious agents is a single conscious agent whose world, *W*, is itself. This appears to be a model of *introspection*, in which introspection emerges, in an intelligible fashion, from the combination of conscious agents.

## Microphysical objects

We have sketched a theory of subjects. Now we use it to sketch a theory of objects, beginning with the microscopic and proceeding to the macroscopic.

The idea is that space-time and objects are among the symbols that conscious agents employ to represent the properties and interactions of conscious agents. Because each agent is finite, but the realm of interacting agents is infinite, the representations of each agent, in terms of space-time and objects, must omit and simplify. Hence the perceptions of each agent must serve as an interface to that infinite realm, not as an isomorphic map.

Interacting conscious agents form dynamical systems, with asymptotic (i.e., long-term) behaviors. We propose that microphysical objects represent asymptotic properties of the dynamics of conscious agents, and that space-time is simply a convenient framework for this representation. Specifically, we observe that the harmonic functions of the space-time chain that is associated with the dynamics of a system of conscious agents are identical to the wave function of a free particle; particles are vibrations not of strings but of interacting conscious agents.

Consider, for concreteness, the system of two conscious agents of section Dynamics of Two Conscious Agents, whose dynamics is governed by the kernel *L* of (17). This dynamics is clearly Markovian, because the change in state depends only on the current state. The *space-time chain* associated to *L* has, by definition, the kernel

(39) $$Q:(E\times \mathbb{N})\times (E⊗2^{\mathbb{N}})\to [0,1],$$

given by

(40) $$Q\left(\left(e,\;n),\;A\times \{m\right\}\right)=\{\begin{array}{cc} L\left(e,\;A\right) & \text{if }m=n+1,\\ 0, & \text{otherwise}, \end{array}$$

where e ∈ E, n,m ∈ ℕ, and A ∈ **E** (Revuz, 1984).

Then it is a theorem (Revuz, 1984) that, if *Q* is quasi-compact (this is true when the state space is finite, as here), the asymptotic dynamics of the Markov chain takes on a cyclical character:

- There are a finite number of invariant events or absorbing sets: once the chain lands in any of these, it stays there forever. And the union of these events exhausts the state space *E*. We will index these events with the letter ρ.
- Each invariant event ρ is partitioned into a finite number *d*_ρ_ of “asymptotic” events, indexed by ρ and by δ = 1, …, *d*_ρ_, so that once the chain enters the asymptotic event δ, it will then proceed, with certainty, to δ + 1, δ + 2, and so on, cyclically around the set of asymptotic events for the invariant event ρ.

Then there is a correspondence between eigenfunctions of *L* and harmonic functions of *Q* (Revuz, 1984, p. 210) We let

(41) $$\lambda_{\rho ,k}=\exp(2i\pi k/d_{\rho }),$$

and

(42) $$f_{\rho ,k}=\sum_{\delta =1}^{d\rho }{\left(\lambda_{\rho ,k}\right)}^{\delta }U_{\rho ,\delta }$$

where *ρ* is the index over the invariant events (i.e., absorbing sets), the variable *k* is an integer modulo *d_ρ_*, and *U_ρ, δ_* is the indicator function of the asymptotic event with index *ρ, δ*. For instance, in the example of section First Example of Asymptotic Behavior, there are 6 absorbing sets, so ρ = 1, 2,…, 6. The first absorbing set has only one state, so *d*_1_ = 1. Similarly, *d*_2_ = 1, *d*_3_ = 2, *d*_4_ = *d*_5_ = *d*_6_ = 4. The function *U*_1,1_ has the value 1 on the state |0000〉 and 0 for all other states; *U*_5,3_ has the value 1 on the state |1100〉 and 0 for all other states.

Then it is a theorem that

(43) $$Lf_{\rho ,k}=\lambda_{\rho ,k}f_{\rho ,k},$$

i.e., that *f_ρ,k_* is an eigenfunction of *L* with eigenvalue λ_ρ,k_, and that

(44) $$g_{\rho ,k}(\cdot ,n)={\left(\lambda_{\rho ,k}\right)}^{-n}f_{\rho ,k},$$

is Q-harmonic (Revuz, 1984). Then, using (41–42), we have

(45) $$\begin{array}{lll} g_{\rho ,k}(\cdot ,n) & = & \exp{\left(2i\pi k/d_{\rho }\right)}^{-n}\sum_{\delta =1}^{d_{\rho }}\exp{\left(2i\pi k/d_{\rho }\right)}^{\delta }U_{\rho ,\delta }\\ & & =\sum_{\delta =1}^{d_{\rho }}\exp(2i\pi k\frac{\delta }{d_{\rho }}-2i\pi k\frac{n}{d_{\rho }})U_{\rho ,\delta }\\ & & =\sum_{\delta =1}^{d_{\rho }}\text{cis}(2\pi \frac{k\delta }{d_{\rho }}-2\pi \frac{kn}{d_{\rho }})U_{\rho ,\delta }\\ & & =\sum_{\delta =1}^{d_{\rho }}\text{cis}(2\pi \frac{\delta }{d_{\rho ,k}}-2\pi \frac{n}{d_{\rho ,k}})U_{\rho ,\delta } \end{array}$$

where *d_ρ,k_* = *d_ρ_ /k*. This is identical in form to the wavefunction of the free particle (Allday, 2009, §7.2.3):

(46) $$\psi (x,t)=A\sum_{x}\text{cis}(2\pi \frac{x}{\lambda }-2\pi \frac{t}{T})|x\rangle$$

This leads us to identify *A* 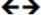 1, *U_ρ,δ_* 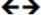
|*x*〉, δ 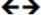 *x*, *n* 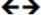 *t*, and *d_ρ,k_* 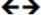 λ = *T*. Then the momentum of the particle is *p* = *h*/*d_ρ,k_* and its energy is *E* = *hc/d_ρ,k_*, where *h* is Planck's constant and *c* is the speed of light.

Thus, we are identifying (1) a wavefunction ψ of the free particle with a harmonic function *g* of a space-time Markov chain of interacting conscious agents, (2) the position basis |*x*〉 of the particle with indicator functions *U_ρ,δ_* of asymptotic events of the agent dynamics, (3) the position index *x* with the asymptotic state index δ, (4) the time parameter *t* with the step parameter *n*, (5) the wavelength λ and period *T* with the number of asymptotic events *d_ρ,k_* in the asymptotic behavior of the agents, and (6) the momentum *p* and energy *E* as functions inversely proportional to *d_ρ,k_*.

Note that wavelength and period are identical here: in these units, the speed of the wave is 1.

This identification is for non-relativistic particles. For the relativistic case we sketch a promising direction to explore, starting with the dynamics of two conscious agents in an undirected join. In this case, the state of the dynamics has six components: *N*_1_, *N*_2_, *X*_1_, *X*_2_, *G*_1_, *G*_2_. We identify these with the generating vectors of a geometric algebra G(2, 4) (Doran and Lasenby, 2003). The components *N*_1_ and *N*_2_ have positive signature, and the remaining have negative signature. G(2, 4) is the conformal geometric algebra for a space-time with signature (1, 3), i.e., the Minkowski space of special relativity. The conformal group includes as a subgroup the Poincare group of space-time translations and rotations; but the full conformal group is needed for most massless relativistic theories, and appears in theories of supersymmetry and supergravity. The Lie group SU(2, 2) is isomorphic to the rotor group of G(2, 4), which provides a connection to the twistor program of Roger Penrose for quantum gravity (Penrose, 2004).

Thus, the idea is to construct a geometric algebra G(2, 4) from the dynamics of two conscious agents, and from this to construct space-time and massless particles. Each time we take an undirected join of two conscious agents, we get a new geometric algebra G(2, 4) with new basis vectors as described above. Thus, we get a nested hierarchy of such geometric algebras from which we can build space-time from the Planck scale up to macroscopic scales. The metric would arise from the channel capacity of the joined agents.

The massive case involves symmetry breaking, and a promising direction to explore here involves hierarchies of stopping times in the Markovian dynamics of conscious agents. The idea is that one system of conscious agents might infrequently interact with another system, an interaction that can be modeled using stopping times. Such interactions can create new conscious agents, using the combination theorems presented earlier, whose “time” is moving more slowly than that of the original systems of agents involved in the combination. This hierarchy of stopping times proceeds all the way up to the slow times of our own conscious experiences as human observers (roughly 10^40^ times slower than the Planck time). The hierarchy of stopping times is linked to a hierarchy of combinations of conscious agents, leading up to the highest level of conscious agents that constitute us, and beyond.

## Objections and replies

Here we summarize helpful feedback from readers of earlier drafts, in the form of objections and replies.

(1) Your definition of conscious agents could equally well-apply to unconscious agents. Thus, your theory says nothing about consciousness.

Even if the definition could apply to unconscious agents, that would not preclude it from applying to consciousness, any more than using the integers to count apples would preclude using them to count oranges.

(2) How can consciousness be cast in a mathematical formalism without losing something essential?

The mathematics does lose something essential, viz., consciousness itself. Similarly, mathematical models of weather also lose something essential, viz., weather itself. A mathematical model of hurricanes won't create rain, and a mathematical model of consciousness won't create consciousness. The math is not the territory. But, properly constructed, mathematics reveals the structure of the territory.

(3) Why do you represent qualia by a probability space X?

Probability spaces can be used, of course, to represent a diverse range of content domains, from the outcomes of coin-flips to the long-term behavior of equity markets. But this does not preclude using probability spaces to represent qualia. A probability space is not itself identical to qualia (or to coin flips or equity markets). To propose that we represent the possible qualia of a conscious agent by a probability space is to propose that qualia convey *information*, since probability and information are (as Shannon showed) transforms of each other. It is also to propose that qualia need not, in general, exhibit other structures, such as metrics or dimensions. Now certain qualia spaces, such as the space of phenomenal colors, do exhibit metrical and dimensional properties. These properties are not precluded. They are allowed but not required. All that is required is that we can meaningfully talk about the information content of qualia.

The qualia *X* of a conscious agent *C* are private, in the sense that no other conscious agent *C_i_* can directly experience *X*. Instead each *C_i_* experiences its own qualia *X_i_*. Thus, the qualia *X* are “inside” the conscious agent *C*. The “outside” for *C* is *W*, or more precisely, *W*-*C*.

(4) A conscious agent should have free will. Where is this modeled in your definition?

The kernel *D* represents the free will choices of the conscious agent *C*. For any particular quale *x* in *X*, the kernel D gives a probability measure on possible actions in the set *G* that the conscious agent might choose to perform. We take this probability measure to represent the free will choice of the conscious agent. Thus, we interpret the probabilities as objective probabilities, i.e., as representing a true nondeterminism in nature. We are inclined to interpret all the other probabilities as subjective, i.e., as reflections of ignorance and degrees of belief.

(5) A conscious agent should have goals and goal-directed behaviors. Where are these modeled in your definition?

Goals and goal-directed behaviors are not in the definition of conscious agent. This allows the possibility of goal-free conscious agents, and reflects the view that goals are not a definitional property of consciousness. However, since one can construct universal Turing machines from dynamical systems of conscious agents, it follows that one can create systems of conscious agents that exhibit goal-directed behaviors. Goals experienced as conscious desires can be represented as elements of a qualia space *X*.

(6) Your theory doesn't reject object permanence, because conscious agents are the “objects” that give rise to our perceptions of size and shape, and those agents are permanent even when we're not looking.

Conscious realism proposes that conscious agents are there even when one is not looking, and thus rejects solipsism. But it also rejects object permanence, viz., the doctrine that 3D space and physical objects exist when they are not perceived. To claim that conscious agents exist unperceived differs from the claim that unconscious objects and space-time exist unperceived.

(7) If our perceptions of space-time and objects don't resemble objective reality, if they're just a species-specific interface, then science is not possible.

The interface theory of perception poses no special problems for science. The normal process of creating theories and testing predictions continues as always. A particularly simple theory, viz., that our perceptions resemble reality, happens to be false. Fine. We can develop other theories of perception and reality, and test them. Science always faces the problem, well-known to philosophers of science, that no collection of data uniquely determines the correct theory. But that makes science a creative and engaging process.

(8) Your proposal that consciousness, rather than physics, is fundamental places consciousness outside of science.

Absolutely not. The onus is on us to provide a mathematically rigorous theory of consciousness, to show how current physics falls out as a special case, and to make new testable predictions beyond those of current physics. To dismiss the physicalist theory that space-time and objects are fundamental is not to reject the methodology of science. It is just to dismiss a specific theory that is false.

(9) You argue that natural selection does not favor true perceptions. But this entails that the reliability of our cognitive faculties is low or inscrutable, and therefore constitutes a defeater for belief in natural selection. See Alvin Plantinga's argument on this (Plantinga, 2002).

Evolutionary games and genetic algorithms demonstrate that natural selection does not, in general, favor true perceptions. But this entails nothing about the reliability of our cognitive faculties more generally. Indeed, selection pressures might favor more accurate logic and mathematics, since these are critical for the proper estimation of the fitness consequences of actions. The selection pressures on each cognitive faculty must be studied individually before conclusions about reliability are drawn.

(10) The undirected join of conscious agents doesn't really solve the problem of combining subjects, because the decision kernel of the combination is just the product of the decision kernels of the two conscious agents that are combined. This product only models two separate agents making separate decisions, not two subjects combined into a single decision-making subject.

It's true that the decision kernel, *D*, of the combination starts out as a product, indicating independent decisions. But as the conscious agents in the combination continue to interact, the decisions become less and less independent. In the asymptotic limit, the decision kernel *D^n^* as *n* → ∞ of the combination cannot, in general, be written as a product. In this limit, the combination now has a single unified decision kernel, not decomposable as a product of the original decision kernels. And yet the two conscious agents in the combination still retain their identities. Thus, the undirected join models a combination process which starts off as little more than the product of the constituent agents but ends up with those agents fully entangled to form a new conscious agent with a genuinely new and integrated decision kernel.

(11) If I have an objection it is that the authors' proposal is maybe not crazy enough. I am with them 100% when they compare neurons to icons on a computer screen. But (if I have understood them correctly) they then go on to attribute absolute existence to consciousness. My own inclination is to propose that consciousness is also just an icon on a computer screen.

Conscious realism is the hypothesis that the objective world *W* consists of conscious agents. The theory of conscious agents is a mathematical theory of consciousness that quantifies over qualia that it assumes really exist. So this theory does assume the existence of consciousness.

However, it does not assume incorrigibility of qualia (to believe one has a quale is to have one) or infallibility about the contents of one's consciousness. Psychophysical studies provide clear evidence against incorrigibility and infallibility [see, e.g., the literature on change blindness (Simons and Rensink, 2005)]. Nor does it assume that the mathematics of conscious agents is itself identical to consciousness; a theory is just a theory.

One might try to interpret the theory of conscious agents as describing a psychophysical monism, in which matter and consciousness are two aspects of a more abstract reality. Such an interpretation, if possible, might still be unpalatable to most physicalists since it entails that dynamical physical properties, such as position, momentum and spin, have definite values only when they are observed.

(12) One problem with section Evolution and Perception is that the authors never define either their notion of Truth, or their notion of Perception. They seem to believe that if you startle at any sound of rustling leaves (as a sort of sensitive predator avoidance system), then when you run from a real predator, you are not in any way in touch with the truth. But this is incorrect.

For sake of brevity, we omitted our definitions of truth and perception from this paper. But they are defined precisely in papers that study the evolution of perception in Monte Carlo simulations of evolutionary games and genetic algorithms (Mark et al., 2010; Hoffman et al., 2013; Marion, 2013; Mark, 2013).

Briefly, we define a *perceptual strategy* as a measurable function (or, more generally, a Markovian kernel) *p:W → X*, where *W* is a measurable space denoting the objective world and *X* is a measurable space denoting an organism's possible perceptions. If *X = W* and *p* is an isomorphism that preserves all structures on *W*, then *p* is a *naïve realist* perceptual strategy. If *X ⊂ W* and *p* is structure preserving on this subset, then *p* is a *strong critical realist* strategy. If *X* need not be a subset of *W* and *p* is structure preserving, then *p* is a *weak critical realist* strategy. If *X* need not be a subset of *W* and *p* need not be structure preserving, then *p* is an *interface* strategy. These strategies form a nested hierarchy: naïve realist strategies are a subset of strong critical realist, which are a subset of weak critical realist, which are a subset of interface.

Naïve realist strategies see all and only the truth. Strong critical realist strategies see some, but in general not all, of the truth. Weak critical realist strategies in general see none of the truth, but the relationships among their perceptions genuinely reflect true relationships in the structure of the objective world *W*. Interface strategies in general see none of the truth, and none of the true relationships in the structure of *W*. Thus, our mathematical formulation of perceptual strategies allows a nuanced exploration of the role of truth in perception.

We let these perceptual strategies compete in hundreds of thousands of evolutionary games in hundreds of thousands of randomly chosen worlds, and find that strategies which see some or all of the truth have a pathetic tendency to go extinct when competing against interface strategies that are tuned to fitness rather than truth. The various truth strategies don't even get a chance to compete in the genetic algorithms, because they are not fit enough even to get on the playing field.

Thus, natural selection favors interface strategies that are tuned to fitness, rather than truth. If an organism with an interface perceptual strategy perceives, say, a predatory lion, then it really does perceive a lion in the same sense that someone having a headache really does have a headache. However, this does not entail that the objective world, *W*, contains an observer-independent lion, any more than a blue rectangular icon on a computer desktop entails that there is a blue rectangular file in the computer. There is something in the objective world *W* that triggers the organism to perceive a lion, but whatever that something is, it almost surely doesn't resemble a lion. A lion is simply a species-specific adaptive symbol, not an insight into objective reality.

(13) In section Evolution and Perception, the authors' argument seems to be: **Argument 1:** (1) Natural selection favors fitness in perceptual systems. (2) Fitness is incompatible with truth. (3) Therefore, natural selection favors perceptions that do not see truth in whole or in part.
  With some minor tweaking, Argument 1 can be made valid. But premise 2 is completely implausible. If a tiger is charging you with lunch on his mind, truth works in the service of fitness. (The authors' treatment here raises the question of why we have perceptual systems at all and not just kaleidoscope eyes. They never address this.)
  The authors would object that premise 2 is too strong. They don't subscribe to premise 2, they would say. They would perhaps hold out for Argument 2:
  **Argument 2:** (1) Natural selection favors fitness in perceptual systems. (2) Fitness need not always coincide with truth. (3) Therefore, natural selection favors perceptions that do not see truth in whole or in part.
  But Argument 2 is not valid and not tweakable into a valid argument. The conclusion is a lot stronger than the premises.
  Worse, any weaker premise doesn't give the authors their needed/wanted radical thesis: Perception is not about truth, it is about having kids. Which they insist must be interpreted as Perception is never about truth, but about having kids. But this interpretation is obviously false. For one thing, if an ancient ancestor of ours (call her, Ug) is successful in having kids, she needs to know the truth: that she has kids! Why? Because Ug needs to take care of them!

We do not use either argument. We simply use Monte Carlo simulations of evolutionary games and genetic algorithms to study the evolution of perceptual strategies (as discussed in Objection 12). We find, empirically, that strategies tuned to truth almost always go extinct, or never even arise, in hundreds of thousands of randomly chosen worlds.

The key to understanding this finding is the distinction between fitness and truth. If *W* denotes the objective world (i.e., the truth), *O* denotes an organism, *S* the state of that organism, and *A* an action of that organism, then one can describe fitness as a function *f*:*W* × *O* × *S* × *A* → ℜ. In other words, fitness depends not only on the objective truth *W*, but also on the organism, its state and the action. Thus, fitness and truth are quite distinct. Only if the fitness function happens to be a monotonic function of some structure in *W*, i.e., so that truth and fitness happen to coincide, will natural selection allow a truth strategy to survive. In the generic case, where truth and fitness diverge, natural selection sends truth strategies to extinction.

To phrase this as an argument of the kind given in the objection we would have **Argument 3**: (1) Natural selection favors fitness in perceptual systems. (2) Truth *generically* diverges from fitness. (3) Therefore, natural selection *generically* favors perceptions that diverge from the truth.

The word *generically* here is a technical term. Some property holds generically if it holds everywhere except on a set of measure zero. So, for instance, the cartesian coordinates (*x*, *y*) of a point in the plane *generically* have a non-zero *y* coordinate. Here we are assuming an unbiased (i.e., uniform) measure on the plane, in which the measure of a set is proportional to its area. Since the set of points with a zero *y* coordinate is the *x*-axis line, and since lines have no area, it follows that generically a point in the plane has a non-zero *y* coordinate. Note, however, that there are *infinitely* many points with a zero *y* coordinate, even though this property is non-generic.

So our argument is that, for an appropriate unbiased measure, fitness functions *generically* diverge from truth, and thus natural selection generically favors perceptions that diverge from truth. This does not entail the stronger conclusion that natural selection *never* favors truth. That conclusion is indeed stronger than our premises and stronger than required for the interface theory of perception. Perhaps *H. sapiens* is lucky and certain aspects of our perceptual evolution has been shaped by a non-generic fitness function that does not diverge from truth. In this case some aspects of our perceptions *might* be shaped to accurately report the truth, in the same sense that your lottery ticket *might* be the winner. But the smart money would bet long odds against it. That's what non-generic means.

The account of the interface theory about Ug's perception of her kids is the same as the account in Objection 12 for the perception of lions. There are no public physical objects. Lions and kids are no more public and observer independent than are headaches. Lions and kids (and space-time itself) are useful species-specific perceptions that have been shaped by natural selection not to report the truth but simply to guide adaptive behavior. We must take them seriously, but it is a logical error to conclude that we must take them literally.

Although our eyes do not report the truth, they are not kaleidoscope eyes because they do report what matters: fitness.

(14) We see then that the authors are caught in version of the Liar: Science shows that perception never cares about truth. Let this statement be *L*. *L* is derived via perception. So is *L* (together with its perceptual base) true or false? If it is one, then it is the other. Contradiction.

This is not our argument. We claim that perception evolved by natural selection. Call this statement *E*. Now *E* is indeed informed by the results of experiments, and thus by our perceptions. We observe, from evolutionary game theory, that one mathematical prediction of *E* is that natural selection generically drives true perceptions to extinction when they compete with perceptions tuned to fitness.

Suppose *E* is true. Then our perceptions evolved by natural selection. This logically entails that our perceptions are generically about fitness rather than truth. Is this a contradiction? Not at all. It is a scientific hypothesis that makes testable predictions. For instance, it predicts that (1) physical objects have no causal powers and (2) physical objects have no dynamical physical properties when they are not observed. These predictions are in fact compatible with quantum theory, and are part of the standard interpretation of quantum theory.

Suppose *E* is false. Then our perceptions did not evolve by natural selection. At present, science has no other theory on offer for the development of our perceptual systems. So, in this case, science cannot at present make an informed prediction about whether our perceptions are true or not. But this is not a logical contradiction.

So there is no liar paradox. And there'd better not be. Science cannot be precluded *a priori* from questioning the veridicality of the perceptions of *H. sapiens*, any more than it can be precluded from questioning the veridicality of the perceptions of other species. David Marr, for instance, argues that “… it is extremely unlikely that the fly has any explicit representation of the visual world around him—no true conception of a surface, for example, but just a few triggers and some specifically fly-centered parameters … ” and that the fly's perceptual information “… is all very subjective” (Marr, 1982, p. 34). Science has no trouble investigating the veridicality of the perceptions of other species and concluding, e.g., in the case of the fly, that they fail to be veridical. Its methods apply equally well to evaluating the veridicality of the perceptions of *H. sapiens* (Koenderink et al., 2010; Koenderink, 2011b, 2013).

(15) Section The Interface Theory of Perception fares no better. Here they say Reality, we learned, departed in important respects from some of our perceptions. This is true. But it is true because other perceptions of ours won out because they were true. E.g., the Earth is not a flat disk or plane.

Other perceptions indeed won out—not because they are true but because they are adaptive in a wider range of contexts. Flat earth is adequate for many everyday activities, but if one wants to circumnavigate the earth by boat then a spherical earth is more adaptive. If one wants to control satellites in orbit or navigate strategic submarines then a spherical earth is inadequate and a more complex model is required.

Perceived 3D space is simply a species-specific perceptual interface, not an insight into objective reality; we have argued for this on evolutionary grounds, and researchers in embodied cognition have arrived at a similar conclusion (Laflaquiere et al., 2013; Terekhov and O'Regan, 2013). Space as modeled in physics extends perceived space via the action of groups, e.g., the Euclidean group, Poincare group, or arbitrary differentiable coordinate transformations (Singh and Hoffman, 2013). Any objects embedded in space, including earth and its 3D shape, are thus descriptions in a species-specific vocabulary, not insights into objective reality.

(16) Also, I don't understand their interface theory of perception. I not only take my icons seriously, but literally: they are icons. I'm prepared to wager the farm on this: they are indeed icons.

We would agree that icons are indeed icons. When I open my eyes and see a red apple, that red apple is indeed an icon of my perceptual interface. When I close my eyes that icon disappears; I see just a mottled gray field. Now some physicalists would like to claim that even when my eyes are closed, an objective red apple still exists, indeed the very red apple that triggered my perceptual interface to have a red apple icon. It is this claim that is generically incorrect, if our perceptual systems evolved by natural selection.

(17) The authors make too much of the Humean idea that the appearance of cause and effect is simply a useful fiction (section The Interface Theory of Perception). They like all mammals and perhaps most animals cannot fail to see causation in the deepest aspects of their lives. The authors believe in causation as deeply as anyone in the world. Why? Because we are all hardwired to see causation. And while it is true that causation goes away at the quantum level, we have no reason to believe that it doesn't really exist at the macro level. These two levels don't live well together, but pretending that there's no such thing as causation is silly, at least it is silly without a lot of argument. Even Hume admitted that causation was perfectly real when he had left his study and went to play backgammon with his friends.

There is indeed good evidence that belief in causation is either innate or learned early in life (Carey, 2009; Keil, 2011). And of course we, the authors, are no exception; we, no less than others, have a psychological penchant toward causal reasoning about the physical world. But, equally, we no less than others have a psychological penchant toward assuming that space, time and physical objects are not merely icons of a species-specific perceptual interface, but are instead real insights into the true nature of objective reality. Science has a habit of correcting our penchants, even those deeply held. Evolutionary games and genetic algorithms convinced us, against our deeply held convictions to the contrary, that perceptions are, almost surely, interfaces not insights; they also convinced us that the appearance of causality among physical objects is a useful fiction.

Perceptual icons do, we propose, *inform* the behavior of the perceiver, and in this sense might be claimed to have causal powers. This sense of causality, however, differs from that typically attributed to physical objects.

Hume's ideas on causation had little influence on us, in part because exegesis of his ideas is controversial, including projectivist, reductionist and realist interpretations (Garrett, 2009).

Our views on causality are consistent with interpretations of quantum theory that abandon microphysical causality, such as the Copenhagen, quantum Bayesian and (arguably) many-worlds interpretations, (Allday, 2009; Fuchs, 2010; Tegmark, 2014). The burden of proof is surely on one who would abandon microphysical causation but still cling to macrophysical causation.

(18) Their treatment of the combination problem is worth reading. There is however a very large problem with their model: It relies on the Cartesian product of *X*_1_ and *X*_2_ (this is right after Conjecture 3). The Cartesian product is not conducive to real combination (this problem is all over mathematics, by the way—mathematicians don't care about it because they only care about high level abstractions). In section Objections and Replies, where they discuss objections to their model, they discuss this very objection (objection 10). Unfortunately, their resolution to this objection is mere handwaving: But as the conscious agents in the combination continue to interact, the decisions become less and less independent. This is mere wishful thinking. The authors have no reason to believe this less and less business and they've given the reader no reason to think this either. In fact, if this less and less business were true, their model wouldn't require the Cartesian product in the first place. Frankly, this objection and their failure to handle it guts their model. In this same paragraph, in the next couple of sentences, the authors just assert (using proof by blatant assertion) that in some undefined limit, a true new conscious entity emerges. This makes the complex presentation of their model otiose. Why not just write a haiku asserting that the combination problem is not a problem?

The limit we speak of (for the emergence of a new combined conscious agent) is the *asymptotic limit*. Asymptotic behavior is a precise technical concept in the theory of Markov chains (see, e.g., Revuz, 1984, chapter 6). We have given, in sections First Example of Asymptotic Behavior and Second Example of Asymptotic Behavior, concrete examples of undirected joins for which, asymptotically, a new combined conscious agent is created that is not just a Cartesian product of the original agents.

Intuitively, the reason that the undirected combination of two agents creates a new agent that is not just a product is that there is feedback between the two agents (this is illustrated in Figure 2). Thus, the decisions and actions of one agent influence those of the other. This influence is not fully felt in the first step of the dynamics, but in the asymptotic limit of the dynamics it completely dominates, carving the state space of the dynamics into various absorbing sets with their own periodic behaviors, in a fashion that is not reducible to a simple product of the original two agents.

The degree to which the new conscious agent is not reducible to a simple product of the original agents can be precisely quantified using, for instance, the measure of *integrated information* developed by Tononi and others (Tononi and Edelman, 1998; Tononi and Spoorns, 2003; Tononi, 2008; Tononi and Koch, 2008; Barrett and Seth, 2011). It is straightforward to compute, for instance, that the new agent in Second Example of Asymptotic Behavior has 2 bits of integrated information, i.e., of new information that is not reducible to that of the two original agents. Thus, there is a precise and quantifiable sense in which the undirected combination of conscious agents creates a new conscious agent with its own new information.

We should note, however, that our use here of Tononi's measure of integrated information does not imply that we endorse his theory of consciousness. Tononi is a reductive functionalist, proposing that consciousness is *identical* to integrated information and that qualia are *identical* to specific informational relationships (Tononi, 2008). Consistent with this view he asserts, for instance, that spectrum inversion is impossible (Tononi, 2008, footnote 8). However, a recent theorem proves that *all* reductive functionalist theories of consciousness are false (Hoffman, 2006). A fortiori, Tononi's theory is false. His measure of integrated information and his analyses of informational relationships are valuable. But his next move, of *identifying* consciousness with integrated information, is provably false. He could fix this by making the weaker claim that consciousness is *caused by* or *results from* integrated information. His theory would no longer be necessarily false. But then he would need to offer a scientific theory about how integrated information causes or gives rise to consciousness. No such theory is currently on offer and, we suspect, no such theory is possible.

(19) The paper explicitly commits a fallacy: it privileges the authors' take on reality while denying that there is any such thing as reality. For example: The authors say “There are no public physical objects. Lions and kids are no more public and observer independent than are headaches. Lions and kids (and space-time itself) are useful species-specific perceptions that have been shaped by natural selection not to report the truth but simply to guide adaptive behavior. We must take them seriously, but it is a logical error to conclude that we must take them literally.”
  Natural selection, which the authors clearly think is the truth, is just as susceptible to their arguments as headaches or truth itself. So by their own reasoning, natural selection is not true; neither are their computer programs/models. So the reader doesn't have to take natural selection or their models either seriously or literally. So their paper is now exposed as self-refuting.

If we indeed proposed a “take on reality while denying that there is any such thing as reality,” we would of course be self-refuting. However, we do not deny that there is any such thing as reality. We cheerfully admit that there is a reality. We simply inquire into the relationship between reality and the perceptions of a particular species, *H. sapiens*. Such inquiry is surely within the purview of science. Moreover all currently accepted theories in science, including evolutionary theory, are appropriate tools for such inquiry.

We find that evolutionary theory entails a low probability that our perceptions are veridical, and thus a high probability that reality is not isomorphic to our perceptions, e.g., of spacetime and objects. This prompts us to propose a new theory of reality, which we have done by defining conscious agents and proposing conscious realism, viz., that reality consists of interacting conscious agents.

This proposal invites us to revisit evolutionary theory itself. The standard formulation of evolutionary theory, i.e., the neo-Darwinian synthesis, is couched in terms of spacetime and objects (such as organisms and genes), which we now take to be a species-specific perceptual representation, not an insight into reality. But we are not forced into self-refutation at this point. It is open to us to formulate a new *generalized* theory of evolution that operates on what we now take to be reality, viz., interacting systems of conscious agents.

A key constraint on our new evolutionary theory is this: When the new evolutionary theory is projected onto the spacetime perceptual interface of *H. sapiens* we must get back the standard evolutionary theory. Thus, we do not take the standard evolutionary theory to be true, but instead to be a “boundary condition” on the new evolutionary theory. Standard evolutionary theory is simply how the new evolutionary theory appears when it is shoehorned into the perceptual framework that *H. sapiens* happens to have.

The process we are describing here is standard procedure in science. We always use our current best theory as a ladder to a better theory, whereupon we can, if necessary, kick away the ladder. However, we needn't take our best theory to be true. It's simply the best ladder we have to our next theory. We are here adopting a philosophy of instrumentalism in regards to scientific theories.

The development of a new generalized theory of evolution is not just an abstract possibility, but is in fact one of our current projects. We are investigating the possibility of keeping the core ideas of standard evolutionary theory that are sometimes referred to as “Universal Darwinism,” ideas that include abstract notions of variation, selection and retention. We plan to apply Universal Darwinism to interacting systems of conscious agents to model their evolution.

The new limited resource that is the source of competition would be *information*, which is the measure we use to quantify the channel capacity of conscious agents. This is a promising direction, since information is equivalent to energy, and information can be converted into energy (Toyabe et al., 2010). Limited energy resources, e.g., in the form of food, are a clear source of competition in standard evolutionary theory.

The new evolutionary theory that we construct should explain why the standard evolutionary theory was a good ladder to the new theory, and why we are justified in kicking away that ladder.

(20) The authors say, “In short, natural selection does not favor perceptual systems that see the truth in whole or in part. Instead, it favors perceptions that are fast, cheap, and tailored to guide behaviors needed to survive and reproduce. Perception is not about truth, it's about having kids.” This is a false dichotomy.

The distinction between truth and fitness, between truth and having more kids, is not a false dichotomy to evolutionary biologists. It is a distinction that is central to their theory. The same objectively true world can have an infinite variety of different fitness functions, corresponding to the variety of organisms, states and actions. A steak that conveys substantial fitness benefits to a hungry lion conveys no benefits to a cow. Each distinct fitness function drives natural selection in a different direction.

(21) In response to the claim that “Your definition of conscious agents could equally well-apply to unconscious agents; thus, your theory says nothing about consciousness.” the authors reply that “Even if the definition could apply to unconscious agents, that would not preclude it from applying to consciousness, any more than using the integers to count apples would preclude using them to count oranges.”
  However, the very fact that the integers can be used to count apples and oranges and peace treaties, etc., is precisely WHY the integers are not a theory of either apples or oranges or peace treaties, etc. The same is true of definitions. If my definition of integer applies equally well to the complex numbers as well as to the integers, then I do not have a definition of integers. Instead I have a definition of complex numbers. So their definition is useless; all they've done is define an agent. Consciousness is not present, except accidentally.

The integers are not considered a theory of peace treaties because they don't have the appropriate mathematical structure to model peace treaties—not because they can be used to count apples and peace treaties.

If one has a mathematical structure that is rich enough to provide a useful theory of some subject, this does not entail that the same structure *cannot* be a useful theory of a different subject. The group SU(3), for instance, models an exact symmetry of quark colors and an approximate symmetry of flavors. No physicist would insist that because SU(3) is a useful theory of quark color it cannot also be a useful theory of flavor. A given Markovian kernel *P* can model a stochastic dynamics, but also a communication channel. The fact that *P* applies to both does not entail that it's a theory of neither.

Similarly, a measurable space *X* might properly represent the conscious color experiences of a human observer, and also the unconscious color judgments of a robotic vision system designed to mimic that observer. No vision scientist would insist that because *X* properly represents the unconscious color judgments of the robotic vision system that therefore *X* cannot model the conscious color experiences of the human observer.

Scientists do not reject a model because it has multiple domains of useful application. They do reject a model if its structure is inappropriate to the domain, or if it makes predictions that are empirically false. These are the appropriate grounds to judge whether the formalism of conscious agents provides an adequate model for consciousness. The possibility that this formalism applies well to other domains does not entail that it cannot apply to consciousness.

## Conclusion

Belief in object permanence commences at 3 months of age and continues for a lifetime. It inclines us to assume that objects exist without subjects to perceive them, and therefore that an account of objects can be given without a prior account of subjects.

However, studies with evolutionary games and genetic algorithms indicate that selection does not favor veridical perceptions, and that therefore the objects of our perceptual experiences are better understood as icons of a species-specific interface rather than as an insight into the objective structure of reality. This requires a fundamental reformulation of the theoretical framework for understanding objects.

This reformulation cannot assume that physical objects have genuine causal powers, nor that space-time is fundamental, since objects and space-time are simply species-specific perceptual adaptions.

If we assume that conscious subjects, rather than unconscious objects, are fundamental, then we must give a mathematically precise theory of such subjects, and show how objects, and indeed all physics, emerges from the theory of conscious subjects. This is, of course, a tall order. We have taken some first steps by (1) proposing the formalism of conscious agents, (2) using that formalism to find solutions to the combination problem of consciousness, and (3) sketching how the asymptotic dynamics of conscious agents might lead to particles and space-time itself. Much work remains to flesh out this account. But if it succeeds, *H. sapiens* might just replace object permanence with objects of consciousness.

### Conflict of interest statement

The authors declare that the research was conducted in the absence of any commercial or financial relationships that could be construed as a potential conflict of interest.
